# Self-Assembling Hydrogels of Naproxen-Conjugated Peptides for Osteoarthritis Treatment

**DOI:** 10.7150/thno.114781

**Published:** 2025-08-11

**Authors:** Lulu Yang, Liang Shao, Puhua Hao, Jiaqi Song, Caiting Meng, Bin Zhu, Hongwen Yu, Wanglin Duan, Xiaohua Fang, Guanying Li, Shichang Liu

**Affiliations:** 1 Department of Spine Surgery, Honghui Hospital, Xi'an Jiaotong University, Xi'an, Shaanxi, 710054, P. R. China.; 2 Department of Biophysics, School of Basic Medical Sciences, Health Science Centre, Xi'an Jiaotong University, Xi'an, Shaanxi, 710061, P. R. China.; 3 The Second Clinical Medical School, Shaanxi University of Chinese Medicine, Xianyang, Shaanxi, 712046, P. R. China.

**Keywords:** drug-peptide conjugate, self-assembly, NSAID, anti-inflammation, osteoarthritis

## Abstract

**Rationale**: Osteoarthritis (OA), a highly prevalent chronic degenerative joint disease, lacks truly effective therapies. Current approaches are limited by systemic toxicity, short drug half-lives, and insufficient efficacy. To address this, we developed an innovative therapeutic approach integrating the pharmacological benefits of nonsteroidal anti-inflammatory drugs (NSAIDs) with the mechanical support and localized delivery advantages of hydrogels.

**Methods**: Various naproxen-peptide conjugates are designed and synthesized. These candidates were screened based on self-assembly behavior, biocompatibility and cyclooxygenase-2 (COX-2) inhibition. NpxFFK underwent further studies including assessment of anti-inflammatory activities and mechanism of action *in vitro*. *In vivo* therapeutic efficacy was evaluated in OA rat model using footprinting assay, micro-CT imaging, MRI imaging, histological staining, and immunohistochemistry. The therapeutic mechanism is explored via RNA sequencing.

**Results**: Among naproxen-peptide conjugates NpxFFX (X = R, H, K, E) tested, NpxFFK demonstrated superior anti-inflammatory efficacy. It self-assembled into a stable hydrogel, exhibiting enhanced retention within the joint cavity and providing sustained anti-inflammatory action. *In vitro* studies revealed that NpxFFK effectively inhibited COX-2 activity, consequently suppressing key inflammatory factors (IL-1β, IL-6, IL-1, and TNF-α). Furthermore, it reprogramed macrophage polarization from M1 toward M2 and promoted chondrocyte proliferation. *In vivo* experiments demonstrate the NpxFFK hydrogel significantly mitigated articular degradation in OA rats, outperforming clinical treatments (naproxen treatment or hyaluronic acid treatment), thereby validating its therapeutic potential for OA.

**Conclusions**: By integrating NSAID pharmacology with self-assembling peptide hydrogel delivery platform, we present a multifunctional strategy that significantly improves upon current OA treatments, underscoring its promise for translational healthcare innovation in OA management.

## Introduction

Osteoarthritis (OA) is a highly prevalent degenerative joint disease characterized by chondrocytes apoptosis, synovial damage, narrowed joint spaces and osteophyte formation 1-3. With aging populations and rising obesity rates, OA incidence is increasing globally. This is particularly evident in China, where the prevalence of primary OA affects up to 46.3% of adult over 40-year-old 4,5. OA causes significant pain, joint dysfunction, reduced quality of life, and numerous complications, imposing a substantial socioeconomic burden on healthcare systems 6. Despite its high prevalence, current OA therapies 7,8 remain palliative, focusing primarily on symptom management. Nonsteroidal anti-inflammatory drugs (NSAIDs), representing the first-line pharmacological intervention, are widely used to alleviate pain and inflammation 9. However, they suffer from systemic toxicity, short half-lives, and poor joint retention 10-12. Intra-articular (IA) injections of hydrogels 13, including hyaluronic acid (HA) 14, offer localized treatment by enhancing synovial fluid viscoelasticity, providing temporary symptom relief and functional improvement 15. Nevertheless, these hydrogels lack inherent anti-inflammatory properties, limiting their clinical efficacy 16-18. Given OA's chronic nature, frequent administration of NSAIDs or HA hydrogel is required, increasing risks of gastrointestinal, renal, and cardiovascular complications 19.

To address the limitations of current OA therapies, we propose an innovative therapeutic approach that integrates the anti-inflammatory benefits of NSAIDs with the mechanical support and sustained-delivery advantages of hydrogels. Naproxen is a potent NSAID that inhibits cyclooxygenase-2 *(*COX-2*)* activity, blocking prostaglandin synthesis from arachidonic acid (AA) and ameliorating the joint inflammatory microenvironment 20-22. Its carboxylic group enables covalent conjugation to hydrogelators that confers the hydrogelation properties 23. To preserve naproxen's efficacy, however, the conjugated hydrogelator must be structural minimal to avoid steric hinderance of its COX-2 binding site 24,25. Ultra-short peptides are emerging as promising building blocks due to their modular design, biocompatibility, and extracellular-mimetic properties 26-28. They self-assemble into low molecular weight hydrogel (LMWH) with tunable nanostructures and mechanical strength, making them ideal for IA delivery. Previous reports have shown that conjugating naproxen to self-assembling peptides did not compromise its *in vitro* COX-2 inhibition 29,30. Notably, conjugating naproxen to D-amino acid-based peptide gelators (e.g. D-phe-D-phe-x, where the x residue represented none, D-lys, or D-tyr, respectively) enhanced COX-2 selectivity toward and stability 31. Structural similarity of naproxen to naphthalene, a commonly peptide capping group, allows intrinsic hydrogelations of naproxen-peptide. For instance, they self-assembled to form stable LMWH 32-34. Despite promising *in vitro* data, the *in vivo* anti-inflammatory efficacy of naproxen-hydrogel systems has been rarely explored, and their integrative benefits for OA therapy remain underexplored.

**Figure 1:**
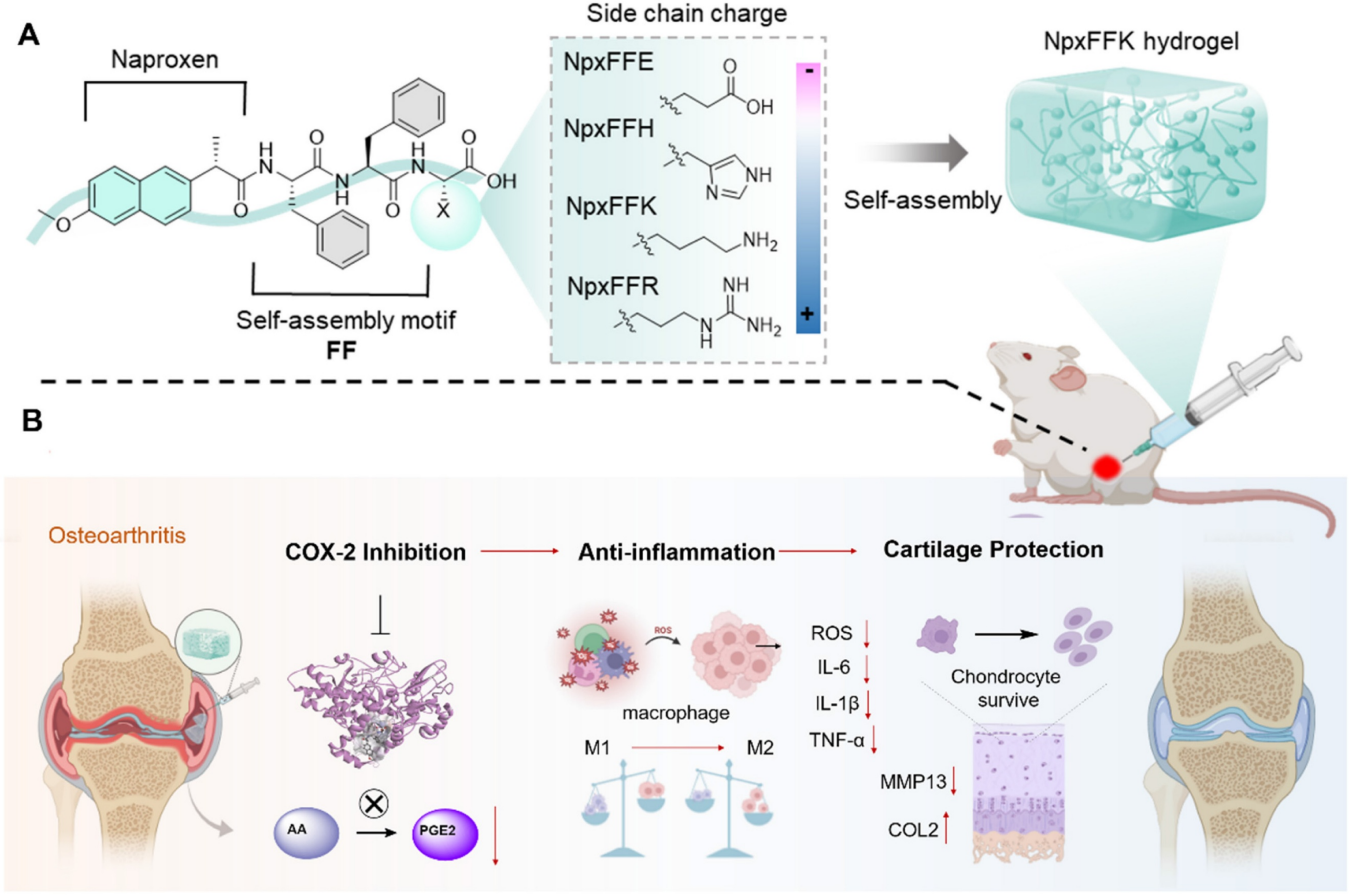
Schematic diagram of the design of naproxen-peptide conjugates as therapeutic hydrogel, and their action mechanism for treating osteoarthritis. (A) Naproxen was conjugated to the N-terminus of self-assembling peptides, obtaining four naproxen-peptide conjugates. Among them, NpxFFK self-assembled into stable low molecular weight hydrogel, suitable of injectable intra-articular administration. (B) Through intra-articular injection, NpxFFK hydrogel blocked COX-2 activity to suppress the conversion of arachidonic acid (AA) to pro-inflammatory prostaglandin E2 (PGE2), consequently downregulating oxidative stress and key inflammatory factors (IL-1β, IL-6, and TNF-α), reprogramming macrophage polarization from pro-inflammatory M1 phenotype to anti-inflammatory M2 phenotype, and promoting chondrocyte proliferations.

In this study, we present comprehensive* in vitro* and* in vivo* evaluation of various naproxen-peptide conjugates, focusing on their self-assembly behavior, mechanical properties, COX-2 inhibitory activity, anti-inflammation effects, and therapeutic efficacy in OA models. Among these tested conjugates, naproxen-Phe-Phe-Lys (NpxFFK) self-assembled into a stable LMWH. This formulation demonstrated significantly enhanced joint retention following IA injection, minimizing systemic exposure and reducing administration frequency while maintaining a sustained anti-inflammatory environment within the joint cavity. The NpxFFK hydrogel exhibited exceptional biocompatibility, potent anti-inflammation effects, and chondroprotective properties in both *in vitro* and *in vivo* models. In OA rat models, IA administration effectively mitigated articular cartilage degradation by suppressing key inflammatory factors and reprogramming macrophages polarization (Figure 1). By combining NSAID pharmacology with a self-assembling peptide hydrogel, we introduce a novel integrative therapeutic approach for efficient OA therapy.

## Results and Discussion

### Design, Synthesis and Structural Characterization

**Figure 2:**
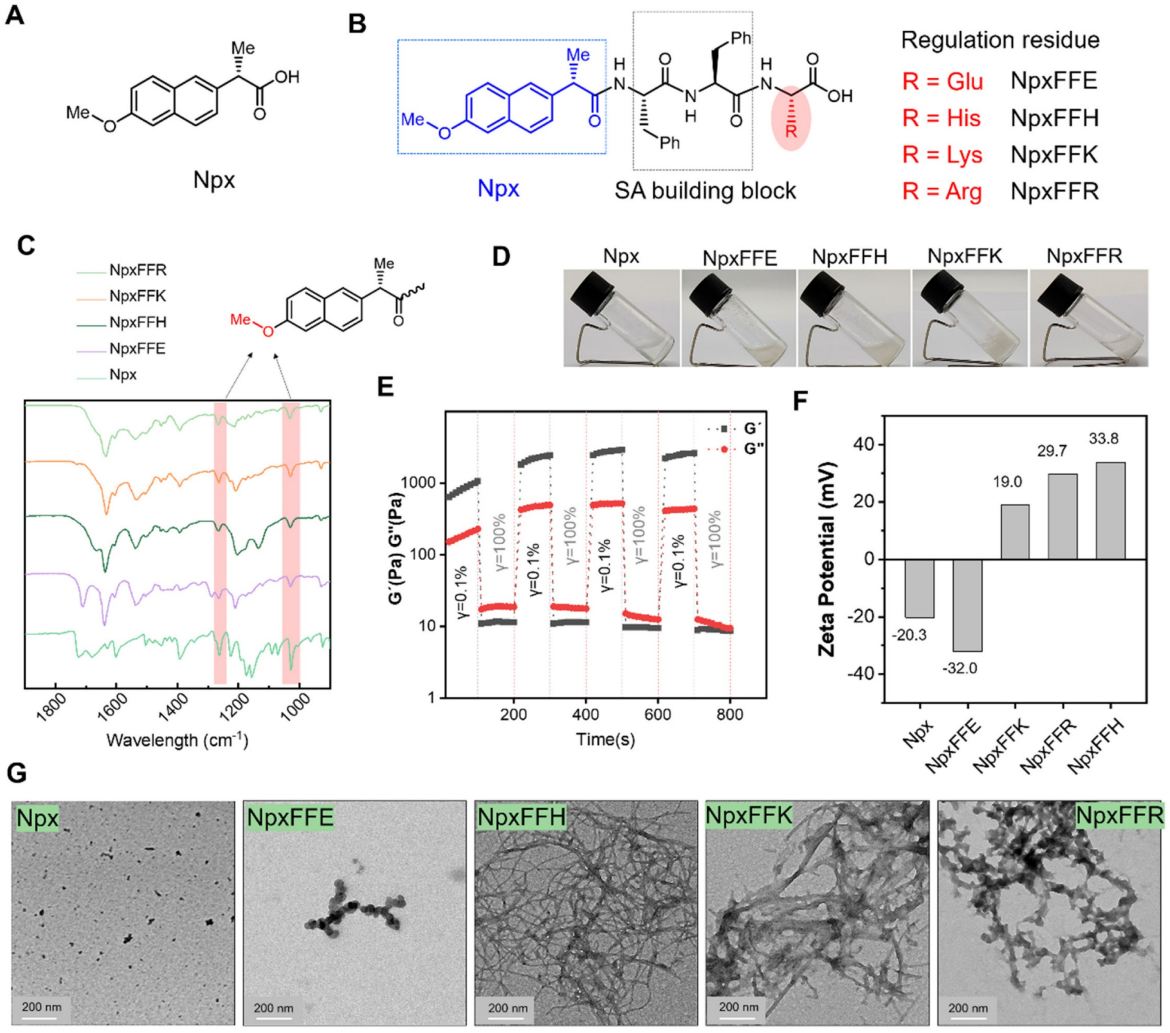
Self-assembly of naproxen-peptide conjugates Chemical structural formula of naproxen (**A**) and naproxen-peptide conjugates (**B**)**.** (**C**) FTIR spectra of NpxFFR (light green), NpxFFK (orange), NpxFFH (dark green), NpxFFE (purple) and Npx (medium spring green). (**D**) Hydrogel formation of Npx-peptide conjugates at 20 mg/mL concentration in ddH_2_O. (**E**) The G' and G'' of the NpxFFK hydrogel from alternate step strain sweep with γ = 0.1% and γ = 1000% at room temperature. (**F**) Zeta potential measurements of 400 µM Npx-peptide conjugates at pH:7.2. (**G**) TEM images of Npx and Npx-peptide conjugates. Scale bars represent 200 nm.

Naproxen, a representative of NSAIDs, faces significant clinical limitations, including severe side effects and rapid clearance from the joint cavity (Figure 2A). Diphenylalanine peptide (FF), a well-established minimal self-assembling motif, can form hydrogels as drug delivery vehicles. Combining FF with naproxen presents an excellent strategy to overcome the weaknesses of naproxen. While prior work by B. Xu demonstrated that NpxFF formed aqueous solution at pH 9.0 with the aid of sonication and heating, which turned into hydrogel when adjusting pH to 4.0 31. This acidic pH dependency restricts its *in vivo* utility. To achieve physiologically relevant hydrogel, we engineered C-terminal charged residues (glutamic acid, histidine, lysine, or arginine) onto NpxFF backbone. The structural modification (1) reduces hydrophobicity to enhance aqueous solubility, (2) enables tunable self-assembly at neutral pH, and (3) modulates COX-2 binding affinity by altering molecular conformation and charge distribution.

Consequently, short peptide conjugates NpxFFE, NpxFFH, NpxFFK, and NpxFFR were prepared via classical solid-phase peptide synthesis (SPPS), with naproxen (Npx) conjugated at the N-terminus (Figure 2B). High-resolution mass spectrometry (Figure S1-S4) and ^1^H NMR analysis (Figure S5-S8) confirmed the molecular identity of all NpxFFX conjugates. Fourier-transform infrared spectroscopy (FT-IR) revealed the characteristic methoxy group vibrations at 1263 cm^-1^ and 1028 cm^-1^, attributed to the C-O-C stretching vibrations (Figure 2C), validating the successful naproxen conjugation.

### Self-Assembly of NpxFFX

We next evaluated the self-assembly capability of these peptide conjugates at physiological pH 7.4. At 20 mg/mL, only NpxFFK formed a stable hydrogel, while NpxFFE, NpxFFH, and NpxFFR yielded suspensions (Figure 2D). Rheological analysis demonstrated that NpxFFK exhibited dominant elastic behavior (G' > G'') acroses 0.1 to 10 Hz frequency sweeps, suggesting stable hydrogel formation. In contrast, NpxFFE, NpxFFH and NpxFFR exhibited weak mechanical properties with G' values declining below detectable limit (< 0.1 Pa) when increasing the frequency. HA exhibited characteristic viscoelastic behavior. These findings confirmed the stable hydrogel formation of NpxFFK (Figure S9A). To assess injectability, we performed stain-recovery tests on NpxFFK hydrogel. Under low strain (γ = 0.1%), As shown in Figure S9B and Figure 2E, the hydrogel maintained structural integrity. At high strain (γ = 100%), G' and G'' sharply decreased due to network disruption. Crucially, immediate recovery occurred upon strain reduction, demonstrating rapid self-healing capability essential for injectable applications.

Zeta potential measurements revealed the surface charges of these naproxen-capped peptides at physiological pH 7.4 (Figure 2F). As anticipated, NpxFFE exhibited a more negative charge (-32mV) than naproxen (-20.3mV), whereas NpxFFH, NpxFFK, and NpxFFR all exhibited positive charges of 33.8 mV, 19 mV, and 29.7 mV, respectively. These macroscopic differences are likely attributed to the charge properties of the peptides. Under physiological conditions, glutamic acid carries a negative charge, while histidine, lysine, and arginine carry positive charges. TEM images revealed that Npx formed uneven aggregates due to relatively low water solubility. Negatively charged NpxFFE self-assembled into spherical particles. NpxFFH and NpxFFK formed reticulated fibrous strands with NpxFFK's strands being slightly thicker than NpxFFH's, and NpxFFR formed flocculent assemblies (Figure 2G).

### Biocompatibility of NpxFFX

**Figure 3:**
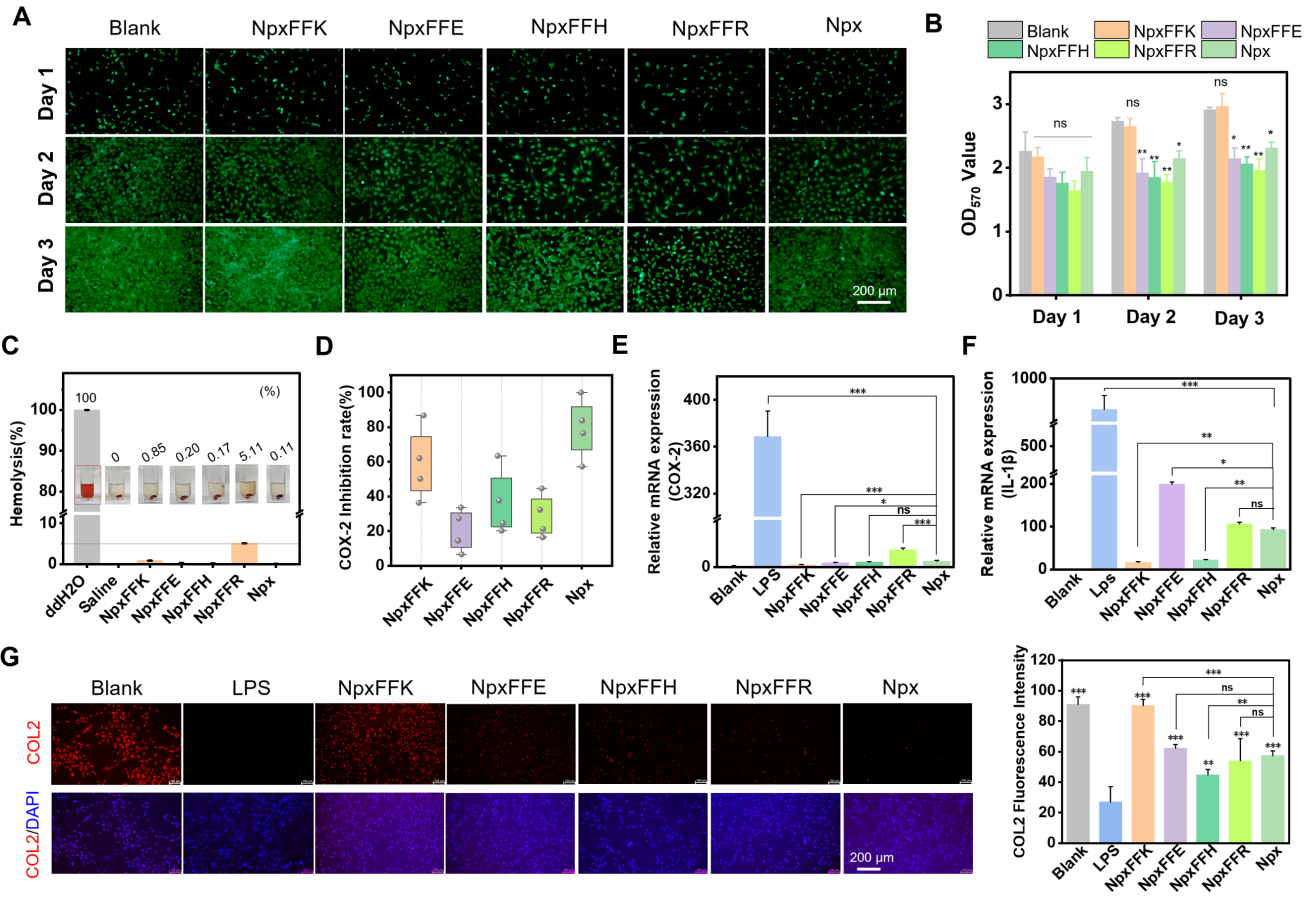
Biosafety assessment of Npx-peptide conjugates**.** Calcein AM staining (**A**) and MTT assay evaluation (**B**) of C28/I2 chondrocytes after treatment with Npx or Npx-peptide conjugates for 1, 2, and 3 days. (**C**) Hemolysis assay of Npx or Npx-peptide conjugates at 400 µM. (**D**) COX-2 inhibition after treatment with 400 µM Npx or Npx-peptide conjugates* in vitro.* (**E**) Relative expression of* COX-2* gene in RAW264.7 cells after treatment with 400 µM Npx or Npx-peptide conjugates for 24 h. (**F**) Relative expression of* IL-1β* gene in RAW264.7 cells after treatment with 400 µM Npx or Npx-peptide conjugates for 24 h. (**G**) Immunofluorescence images and quantitative fluorescence intensities of COL2 in C28/I2 chondrocytes after treatment with 400 µM Npx or Npx-peptide conjugates for 24 h. Scale bars in panel **A** and **G** represent 200 µm. n = 3, ****p < 0.0001, ***p < 0.001, **p < 0.01, *p < 0.05, ns represents non-significance, respectively. The blank groups in panel **A-B** referred to the cells treated with growing media; the blank groups in panel **E-G** referred to the cells without stimulation of LPS.

As a potential hydrogel-based OA treatment, the formulation must possess excellent biocompatibility. In this study, we used the MTT assay to evaluate the cytotoxicity of naproxen-peptide conjugates on C28/I2 chondrocytes 35. As shown in Figure S10, these conjugates exhibited no cytotoxicity to C28/I2 chondrocytes at concentrations below 1 mM. When the concentration exceeded 1 mM, cell viability dropped below 80%, with NpxFFH showing a significant reduction to as low as 20%. Live-dead staining confirmed minimal cytotoxicity at 400 µM (Figure S11). We further evaluated their effects on chondrocytes proliferation using Calcein-AM staining (Figure 3A) and MTT quantification (Figure 3B). All cell groups remained viable after a 3-day incubation. Compared to the non-treated control, the NpxFFK group promoted chondrocyte proliferation, while the proliferation decreased over time in the NpxFFE, NpxFFH, NpxFFR, and Npx groups. Subsequently, we conducted hemolysis assay to assess peptides toxicity (Figure 3C). Red blood cells in ddH₂O showed hemoglobin release (red supernatant), indicating hemolysis. In contrast, supernatant from peptide-treated samples in saline remained clear, with no significant red discoloration, demonstrating minimal hemolysis. The hemolysis rate for NpxFFR reached 5%, the accepted threshold for minimal hemolytic activity. Rates for NpxFFE, NpxFFH, NpxFFK, and Npx were consistently below 5%, confirming their good *in vivo* biocompatibility.

It is established that Npx inhibits COX-2, thereby suppressing production of the inflammatory mediator prostaglandin E (PGE2). This ultimately achieves anti-inflammatory effects by downregulating pro-inflammatory factors, such as IL-1β, IL-1, IL-6, and TNF-α*.* To identify the optimal self-assembling peptide for anti-inflammatory activity, we evaluated COX-2 inhibition by Npx-peptide conjugates (Figure 3D). Among these, NpxFFK showed the most significant COX-2 inhibitory effect, though slightly lower than Npx alone. In addition to the activity inhibition, NpxFFK significantly reduced* COX-2* mRNA expression in LPS-stimulated RAW264.7 cells compared to Npx (Figure 3E), suggesting enhanced anti-inflammatory efficacy. Consequently, we assessed the anti-inflammatory effect of these conjugates. Pro-inflammatory factors such as interleukin-1 beta (*IL-1β*), tumor necrosis factor-alpha (*TNF-α*), and interleukin-6 (*IL-6*) were significantly upregulated in LPS-stimulated Raw264.7 cells (Figure 3F, S12). Treatment with Npx-peptide conjugates markedly downregulated these factors, with NpxFFK showing the strongest suppression due to its potent COX-2 inhibition. NpxFFK treatment effectively restored collagen 2 (COL2) expression in chondrocytes compared to other groups (Figure 3G), suggesting its significant potential for promoting collagen regeneration.

The screening studies on the self-assembly behaviors, biocompatibility, COX-2 inhibition and anti-inflammatory activities of these Npx-peptide conjugates demonstrated that NpxFFK is the most potent candidate. Therefore, we next investigated its molecular mechanism and its therapeutic efficacy in OA intervention both *in vitro* and *in vivo*.

### Anti-inflammatory Efficacy of NpxFFK

**Figure 4:**
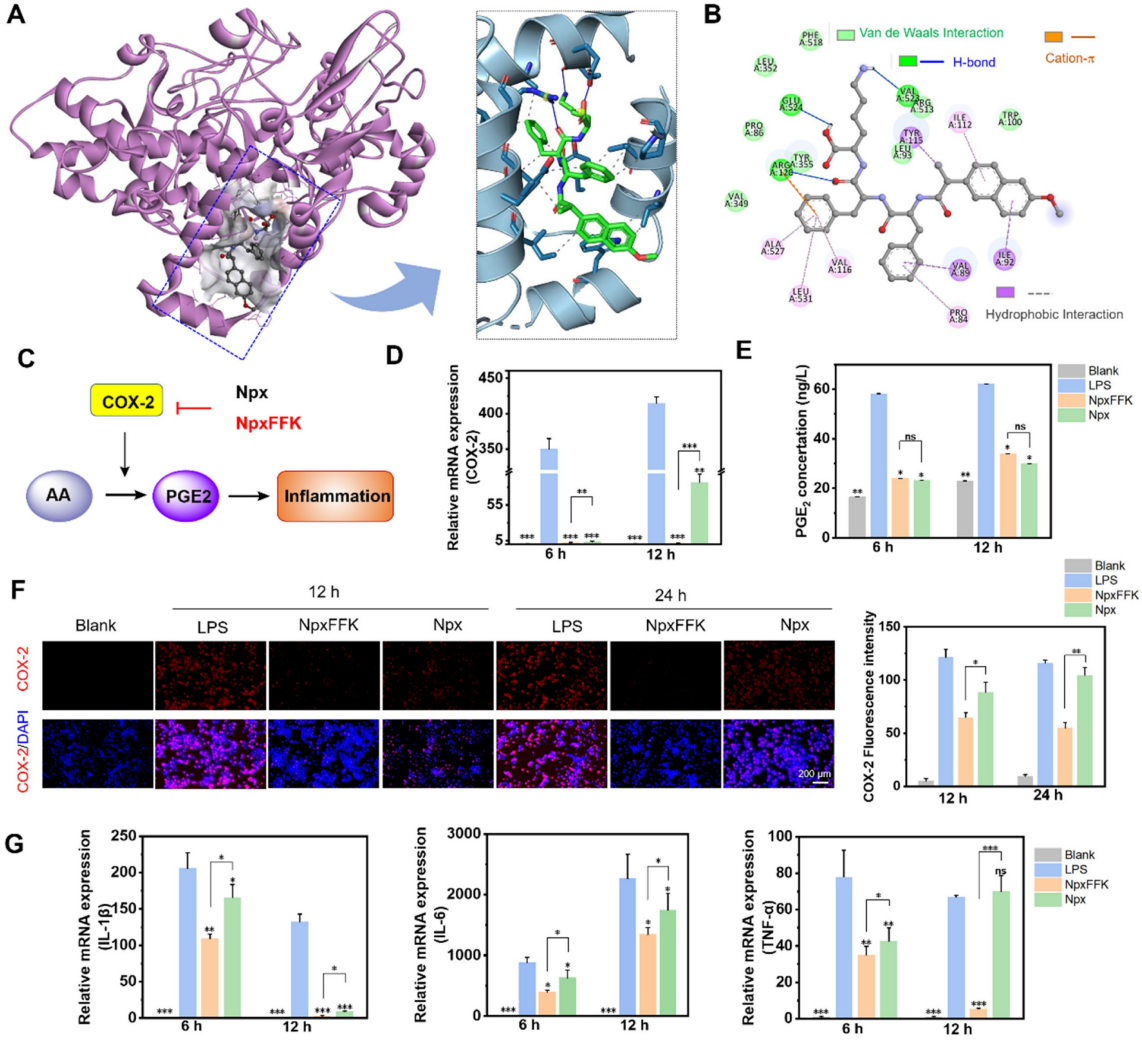
COX-2 inhibition of NpxFFK. (**A**) molecular docking of COX-2 (PDB ID: 6*COX*) with NpxFFK. (**B**) Binding of NpxFFK with amino acid residues in the active pocket of COX-2. (**C**) Schematic representation of the anti-inflammatory mechanism of Npx and the peptide NpxFFK. (**D**) *COX-2* gene expression in LPS-stimulated RAW264.7 cells after treatment with 400 µM Npx or NpxFFK for 6 h, 12 h (n = 3). (**E**) ELISA quantification of PGE2 inflammatory factors in LPS-stimulated RAW264.7 cells after treatment with 400 µM Npx or NpxFFK for 6 h, 12 h (n = 3). (**F**) Immunofluorescence imaging of COX-2 in LPS-stimulated RAW264.7 cells after treatment with 400 µM Npx or NpxFFK for 12 h or 24 h. Scale bars represent 200 µm. (**G**) RT-qPCR quantification of *IL-1β*, *IL-6*, and *TNF-α* expression in LPS-stimulated RAW264.7 cells after treatment with 400 µM Npx or NpxFFK for 6 h or 12 h. n = 3, ***p < 0.001, **p < 0.01, *p < 0.05, ns represents non-significance, respectively. The blank groups in panel **D-G** referred to the RAW264.7 cells without stimulation of LPS.

Molecular docking (Figure 4A) was conducted to verify the binding affinity between NpxFFK and COX-2. The structure of COX-2 was obtained from the Protein Data Bank (PDB ID: 6*COX*) 36 and for docking simulaton with NpxFFK. Similar to naproxen, NpxFFK bound to COX-2 within the classic active pocket. The carboxylic group of Npx interacted with Arg-120 and Tyr-355 residues of COX-2 (Figure S13). These same residues were also involved in the interactions with NpxFFK, suggesting NpxFFK inhibited COX-2 through a mechanism similar to that of Npx. Additionally, the Npx moiety in NpxFFK engaged in hydrophobic interactions with the Ile-92, Ile-112 and Tyr115 residues in COX-2. The conjugated FFK peptide sequence contributed further binding interactions within the active site, including hydrogen bonding, cation-π interaction and hydrophobic contacts, respectively (Figure 4B). The calculated binding free energy for NpxFFK-COX-2 was -9.06 kcal/mol, demonstrating comparable binding affinity to Npx-COX-2 complex (-9.36 kcal/mol). The inhibition constant (Ki) of NpxFFK was determined to be 227.9 nM, slightly higher than that of Npx (136.7 nM, Table S1).

COX-2 is upregulated during inflammation and catalyzes the sequential conversion of arachidonic acid (AA) to prostaglandin G2 (PGG2), then to PGH2, and ultimately to pro-inflammatory PGE2 (Figure 4C). Our studies demonstrated that NpxFFK exerted potent anti-inflammatory effects through COX-2 inhibition, thereby suppressing downstream PGE2 production and inflammatory cytokines expression (*IL-1β*, *IL-1,* and *IL-6*). To further investigate the anti-inflammatory effects of NpxFFK at the cellular level, we used LPS-stimulated RAW264.7 macrophages (1 µg/mL LPS), which showed a significantly elevated *COX-2* gene expression. Treatment with NpxFFK or Npx downregulated COX-2 expression at both the gene level (Figure 4D) and the protein level (Figure 4F). Notably, NpxFFK demonstrated stronger and more sustained COX-2 inhibition than Npx, particularly at longer incubation time (12 hour for PCR evaluation and 24 h for immunofluorescence staining). Consistent with COX-2 inhibition, both treatments reduced PGE2 production (Figure 4E). Furthermore, NpxFFK treatment significantly suppressed the expression levels of key inflammatory factors such as *IL-1β, TNF-α* and* IL-6*, with a more pronounced suppressive effect compared to Npx treatment (Figure 4G). These results collectively indicated that NpxFFK possesses superior and longer-lasting anti-inflammatory effects compared to free Npx.

**Figure 5:**
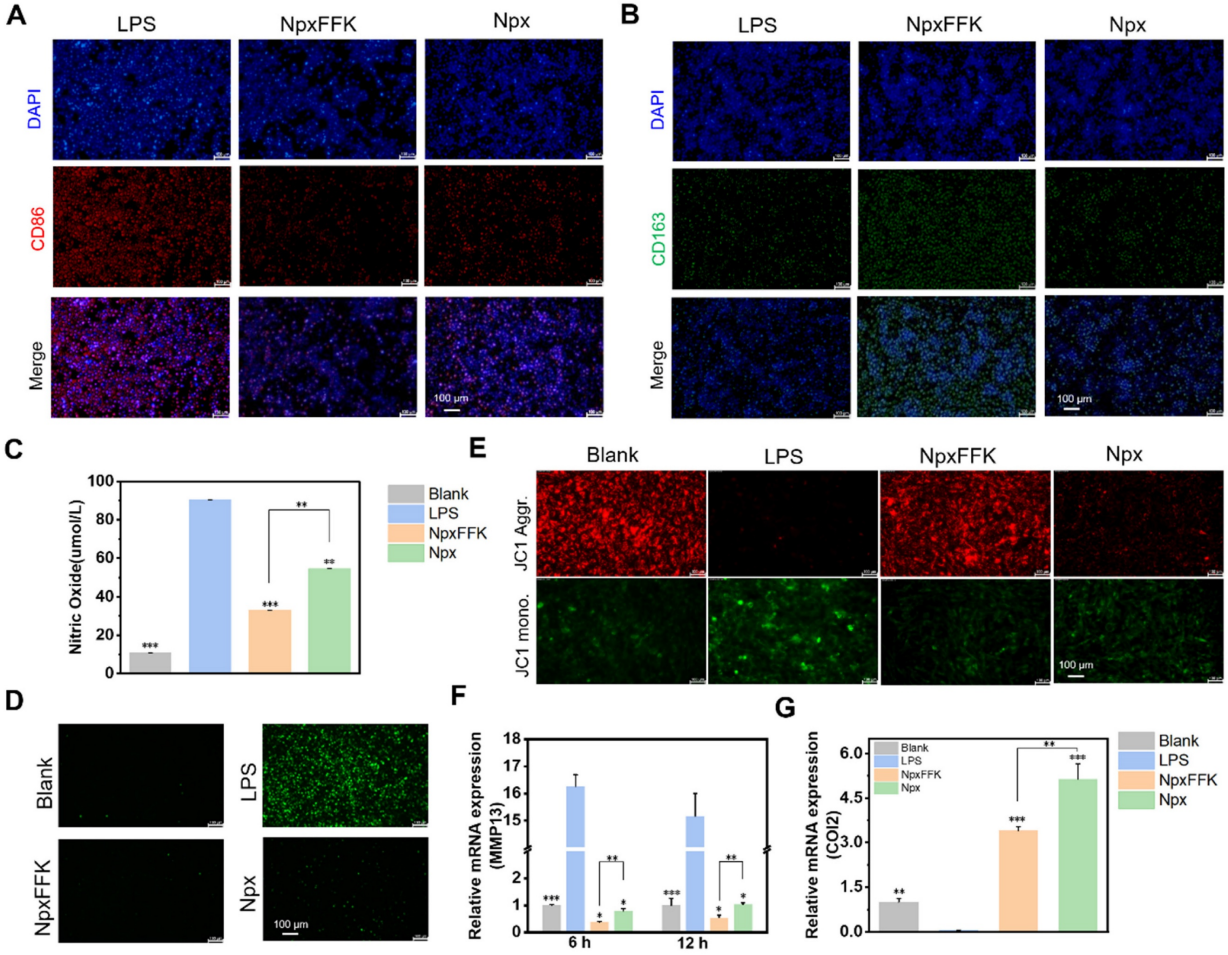
Anti-inflammatory efficacy of NpxFFK. Immunofluorescence image of M1 macrophage (**A**) and M2 macrophage (**B**). Scale bars represent 100 μm. (**C**) Concentration of nitric oxide produced by LPS-stimulated RAW264.7 cells after treatment with 400 µM Npx or NpxFFK for 12 h (n = 3). (**D**) Fluorescence images of intracellular ROS levels in LPS-stimulated RAW264.7 cells after treatment with 400 µM Npx or NpxFFK for 12 h. (**E**) JC-1 staining of mitochondria in LPS-stimulated C28/I2 cells after treatment with 400 µM Npx or NpxFFK for 12 h. (**F**) RT-qPCR quantification of *MMP13* level in LPS-stimulated C28/I2 cells after treatment with 400 µM Npx or NpxFFK for 6 h or 12 h. (**G**) RT-qPCR quantification of *COL2* level in LPS-stimulated C28/I2 cells after treatment with 400 µM Npx or NpxFFK for 12 h. Scale bars in all images panels represent 100 µm. n = 3, ***p < 0.001, **p < 0.01, *p < 0.05, respectively. The blank groups in panel **D-G** referred to the RAW264.7 cells without stimulation of LPS.

Macrophages exhibit two functional polarization states, pro-inflammatory M1 and anti-inflammatory M2, which are crucial for balancing inflammatory response and tissue repair processes. M1 macrophages mediate pathogen defense through secretion of inflammatory cytokines, while M2 macrophages promote tissue repair and inflammation resolution 37. To assess the impact of NpxFFK on macrophage polarization, we performed immunofluorescent analysis of Raw264.7 cells using specific surface markers (CD86 as M1 phenotype marker and CD163 as M2 phenotype marker). As shown in Figures 5A-5B and S14, NpxFFK treatment significantly increased CD163 expression and decreased CD86 expression compared to the LPS stimulation. These results suggested NpxFFK promotes macrophage polarization toward the anti-inflammatory M2 phenotype while suppressing pro- inflammatory M1 phenotype, further confirming its potent anti-inflammatory properties. Oxidative stress caused by inflammatory stimuli exacerbates macrophage-mediated inflammatory responses through the initiation of pro-inflammatory pathways, which can promote chondrocyte apoptosis, enhance catabolic activity, and ultimately establish a destructive cycle of persistent inflammation and cartilage degradation 38. The antioxidant capacity of NpxFFK was evaluated. Npx itself exhibited neglectable radical scavenge activity. NpxFFK showed moderate but significantly antioxidant activity, effectively scavenging both DPPH and ABTS^+^ radicals* in vitro* (Figure S15). LPS stimulation on RAW264.7 cells caused massive nitric oxide (NO) production due to upregulation of inducible nitric oxide synthase (iNOS) under inflammation 39, as well as elevated levels of intracellular ROS. Notably, NpxFFK treatment significantly reduced both NO production and intracellular ROS level, with greater efficacy than free Npx (Figure 5C-D, and S16).

To assess the chondroprotective potential of NpxFFK, we established an* in vitro* inflammation model by treating C28/I2 chondrocytes with the condition media collected from LPS-stimulated Raw264.7 cells. As shown in Figure 5E-G, the inflammatory stress reduced the mitochondrial membrane potentials of chondrocytes. Moreover, it accelerated catabolic enzymes secretion such as matrix metalloproteinase-13 (MMP-13), subsequently degrading cartilage matrix such as type-II collagen (COL2). NpxFFK treatment significantly restored the mitochondrial membrane potentials of C28/I2 cells, downregulated MMP13 gene expression, and enhanced *COL2* gene expression. These results suggested that NpxFFK effectively protects chondrocytes against inflammation-induced mitochondrial damage and MMP-mediated extracellular matrix degradation, showing potential therapeutic efficacy in cartilage repairing.

### Therapeutic Efficacy of Peptide Hydrogel NpxFFK in Osteoarthritis

**Figure 6:**
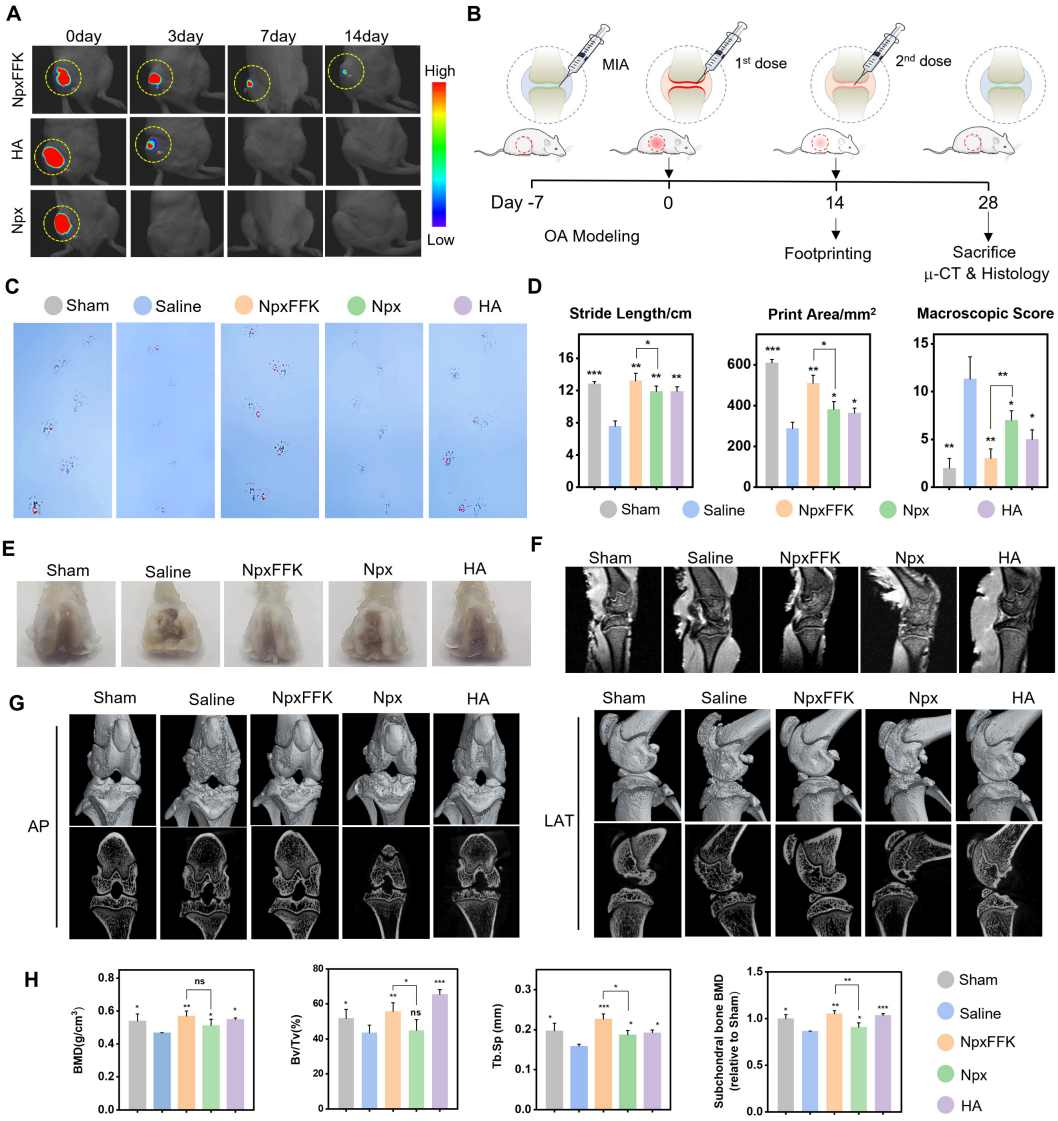
*In vivo* therapeutic assessment of NpxFFK. (**A**) *In vivo* imaging of SD rat after intra-articular injection with Npx solution, HA or NpxFFK hydrogels co-loaded with IR783 dye. (**B**) Schematic representation of OA modeling and OA management in SD rats. (**C**) Footprinting analysis of osteoarthritic rats after treatment with 100 mg/kg Npx, NpxFFK or HA for 2 weeks. (**D**) Quantitative analysis of step lengths, print areas and osteoarticular Macroscopic Scores in footprinting assay. (**E**) Representative images of the articular surfaces in the rats after treatment with 100 mg/kg Npx, NpxFFK or HA for 4 weeks. Representative MRI images (**F**) and micro-CT images (**G**) of the joints in rats after treatment with 100 mg/kg Npx, NpxFFK or HA for 4 weeks. AP: Anterior (Ventral)/Posterior (Dorsal); LAT: Lateral. **(H)** Quantitative analysis of bone mineral density (BMD); BV/TV spicule volume/spicule bone volume; Tb.sp and subchondral bone density in micro-CT images. n = 3, ***p < 0.001, **p < 0.01, *p < 0.05, respectively.

Prior to evaluating therapeutic efficacy for OA treatment, we assessed drug retention in the joint cavity. The NpxFFK hydrogel demonstrated slow degradation rate in PBS, with approximately 50% remaining after 14 days (Figure S17). To track *in vivo* retention, we embedded an NIR-emitting dye IR783 in NpxFFK hydrogel or mixed it with Npx solution in saline for intra-articular injection, monitoring fluorescent signals over time. For clinical comparison, we also tested hyaluronic acid (HA), a standard OA therapy. As shown in Figure 6A and S18, the IR783/Npx solution group showed complete fluorescence disappearance by day 3, with no detectable signal remaining in the knee joint. IR783 embedded in HA exhibited significant signal reduction by day 7, while IR783 in NpxFFK hydrogel maintained detectable fluorescence in the joint cavity for up to 14 days. These results demonstrate the superior sustained retention capability of NpxFFK hydrogel, providing the prolonged presence needed for continuous anti-inflammatory drug action.

Given the NpxFFK hydrogel's demonstrated antioxidant, anti-inflammatory, and chondroprotective properties *in vitro*, we proceeded to evaluate its therapeutic potential in a rat arthritis model. We induced arthritis through IA injection of sodium iodoacetate (MIA), a well-established method valued for its simplicity and rapid model development. Successful arthritis induction was confirmed seven days post-MIA injection. Animals were then randomly divided into four treatment groups: normal saline (control), NpxFFK hydrogel, free Npx, and HA. All treatments were administered via IA injection every two weeks for 28 days to systematically assess therapeutic efficacy (Figure 6B).

To assess the effects of Npx and NpxFFK hydrogel on arthritis-associated pain, we employed footprints analysis using blue and red ink to distinguish forepaw (blue) and hindpaw (red) impressions (Figure 6C). The stride lengths and printing areas were measured for quantitative analysis (Figure 6D). The sham group demonstrated a baseline stride length of 12.83 cm, while the saline-treated OA model group showed significant shortening to 7.57 cm. Npx and HA treatments yielded intermediate improvements (9.42 cm and 10.15 cm, respectively). Notably, the NpxFFK hydrogel group achieved a stride length of 13.20 cm statistically comparable to sham controls, indicating substantial recovery from pain-induced gait impairment. We concurrently assessed hindpaw contact area, a sensitive indicator of arthritic pain (reduced contact reflects tiptoe behavior). The saline group showed marked contact area reduction versus sham controls. While Npx and HA provided partial improvement, NpxFFK hydrogel completely restored contact area to sham levels (Figure 6D). These results demonstrated that NpxFFK hydrogel surpasses both Npx and HA in restoring normal stride length and paw contact area. This superior performance likely stems from its sustained drug release properties, maintaining effective anti-inflammatory and analgesic concentrations at the injury site.

After completing the initial 14-day treatment period, we administered a second dose of saline, NpxFFK hydrogel, free Npx, or HA hydrogel for an additional 14 days. Following this extended treatment, we euthanized the rats and collected knee joints for comprehensive anatomical, histological evaluation and RNA sequencing. Macroscopic examination of femoral specimens (Figure 6E) revealed severe cartilage destruction in saline-treated controls, with extensive erosion of the femoral cartilage layer and visible exposure of subchondral bone in localized defect areas - hallmarks of advanced OA pathology. Both NpxFFK hydrogel and HA treatments showed marked reductions in cartilage damage severity compared to saline controls. MRI images revealed that rats upon treatments with NpxFFK hydrogel or HA maintained complete knee cartilage architecture comparable to sham controls (Figure 6F). However, micro-CT analysis revealed important differences: while HA treatment showed residual cartilage erosion and joint cavity damage, NpxFFK hydrogel effectively prevented bone destruction and osteophyte formation (Figure 6G). The microstructures of the subchondral trabecular bone were further assessed by measuring bone mineral density (BMD), bone volume fraction (BV/TV), trabecular separation (Tb.SP), and trabecular number (Tb.N). These measurements (Figure 6H) confirmed that NpxFFK treatment significantly improved all bone quality metrics compared to other treatment groups. Collectively, our findings demonstrate that NpxFFK hydrogel produces superior therapeutic outcomes in this OA model, outperforming both free Npx and HA treatments across multiple assessment modalities.

**Figure 7:**
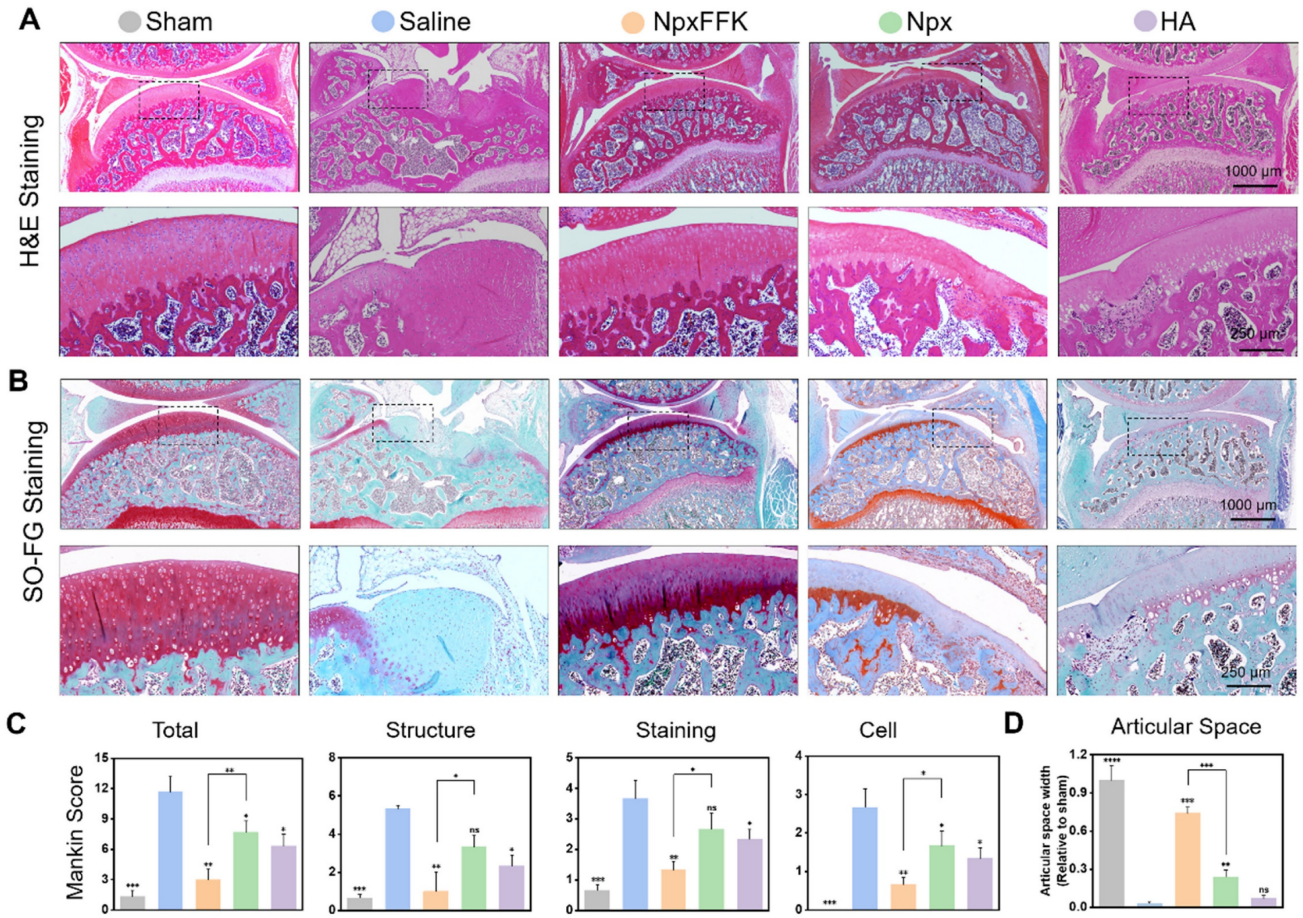
Histological evaluation of OA therapy. H&E staining (**A**) and Safranin O and Fast Green staining (**B**) of the joint sections in rats after treatment with 100 mg/kg Npx, NpxFFK or HA for 4 weeks. (**C**) Mankin scoring incuding the total scores and Mankin Score presented as cartilage structure (Structure), cellular abnormalities (Cell), and matrix staining (Staining). (**D**) Articular spacing width of the joint sections in rats after treatment with 100 mg/kg Npx, NpxFFK or HA for 4 weeks. n = 3, ***p < 0.001, **p < 0.01, *p < 0.05, ns represents non-significance, respectively.

Histological analysis using hematoxylin and eosin (H&E) staining (Figure 7A) and Safranin O-Fast green staining (Figure 7B) further corroborated these findings. The saline-treated control group demonstrated characteristic OA pathology, showing substantial cartilage erosion, extracellular matrix breakdown, and disrupted chondrocyte organization. In striking contrast, the NpxFFK treatment group maintained well-preserved cartilage architecture with smooth articular surfaces and minimal signs of chondrocyte damage, demonstrating exceptional cartilage protection. Notably, the articular cartilage in the NpxFFK group has a better structure and smoother surface than that in the Npx and HA injection group. Safranin O-Fast green staining patterns showed particularly uniform proteoglycan distribution throughout the cartilage matrix of NpxFFK-treated joints, indicating active tissue repair processes. Quantitative assessment using the modified Mankin scoring system 40 objectively demonstrated the NpxFFK group's superior efficacy in mitigating OA progression compared to free Npx treatment (Figure 7C). Furthermore, joint space measurements confirmed that NpxFFK hydrogel treatment effectively prevented pathological joint space narrowing, outperforming both Npx and HA therapeutic approaches (Figure 7D).

**Figure 8:**
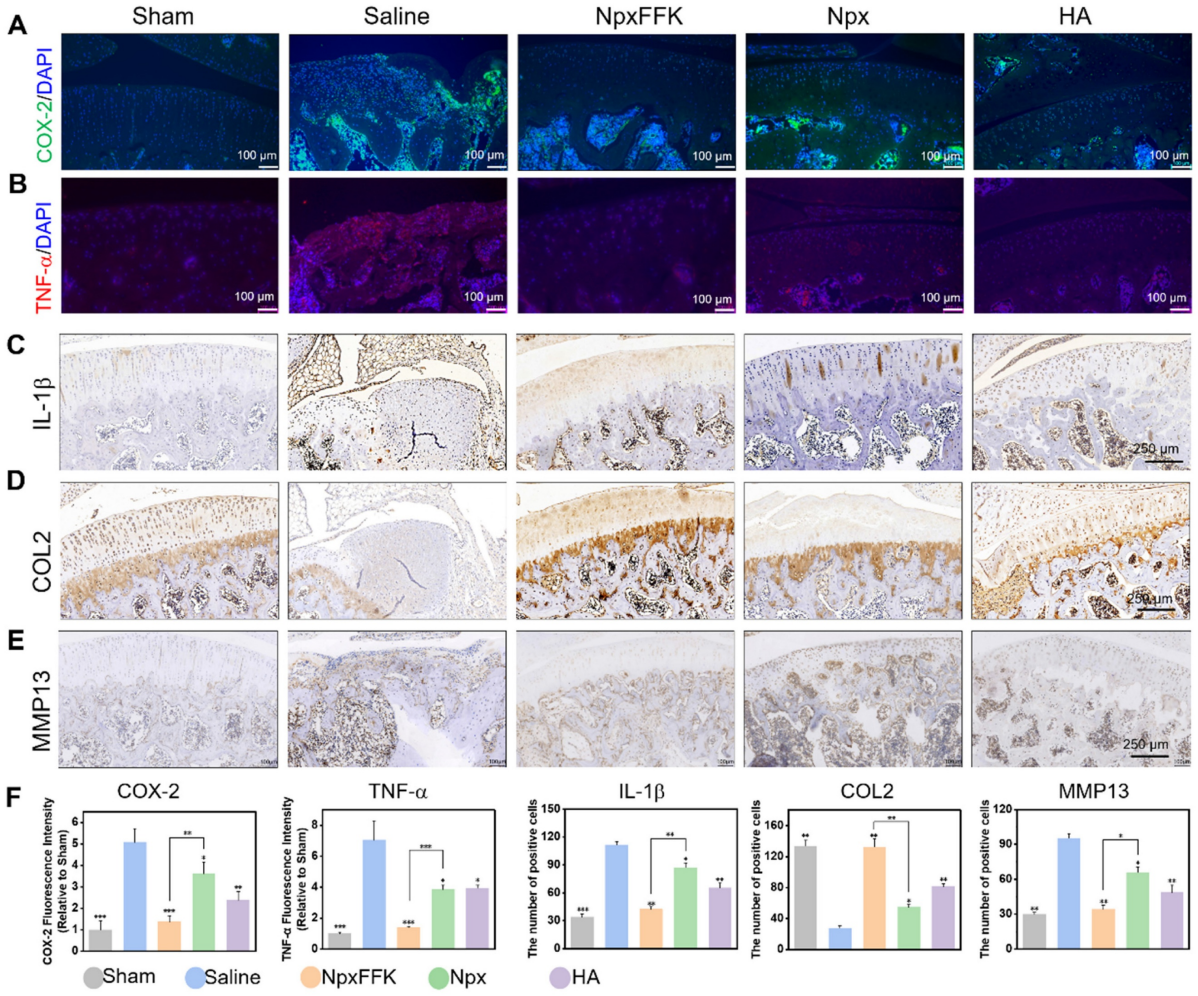
Immunohistochemical evaluation of OA therapy. Representative fluorescent images of COX-2 (**A**) or TNF-α (**B**) in the joint sections after treatment with 100 mg/kg Npx, NpxFFK or HA for 4 weeks. Representative immunohistochemical images of IL-1β (**C**), COL2 (**D**), or MMP13 (**E**) in the joint sections after treatment with 100 mg/kg Npx, NpxFFK or HA for 4 weeks. (F) Quantitative analysis of immunofluorescences intensities in panel **A** and **B**, or quantitative analysis of immunohistochemical staining areas in panel **C**-**E**.

COX-2 is upregulated in OA and rapidly responds to proinflammatory stimuli, with cytokines like TNF-*α* and IL-1*β* driving subsequent cartilage breakdown. Immunofluorescence and immunohistochemical analyses revealed substantially elevated COX-2, TNF-α, IL-1β levels in saline-treated OA controls, confirming characteristic inflammatory pathology (Figure 8A-F). The NpxFFK hydrogel treatment completely suppressed COX*-2* level. Treatment with NpxFFK, Npx, and HA significantly reduced the expression of COX-2 (Figure 8A) and pro-inflammatory cytokines TNF-α (Figure 8B) and IL-1β (Figure 8C), with NpxFFK showing the most pronounced suppression consistent. This* in vivo* anti-inflammatory effect was further supported by macrophage polarization analysis, where NpxFFK treatment significantly decreased pro-inflammatory M1 phenotype while promoting the anti-inflammatory M2 phenotype (Figure S19), corroborating its anti-inflammatory effects. Type II collagen (COL2), the fundamental structural component of cartilage extracellular matrix, is essential for maintaining tissue integrity. Immunohistochemistry (Figures 8D and 8F) showed severe depletion of COL2 in saline-treated OA joints, indicating ECM synthesis impairment. In contrast, NpxFFK treatment markedly restored COL2 expression, demonstrating enhanced cartilage repair capacity. Furthermore, NpxFFK hydrogel significantly suppressed MMP13 expression in joint tissues compared to saline controls (Figures 8E-F), protecting against matrix degradation. Blood tests revealed that upon NpxFFK hydrogel treatment, hematological parameters of the rats remained within normal ranges (Figure S20). H&E staining of major organs including hearts, livers, spleens, lungs, kidneys, and stomachs demonstrated no signs of chronic inflammation or tissue damage (Figure S21). These findings collectively established NpxFFK hydrogel as both highly effective and biocompatible for long-term OA treatment.

### Therapeutic Mechanism Evaluation through RNA Sequencing

**Figure 9:**
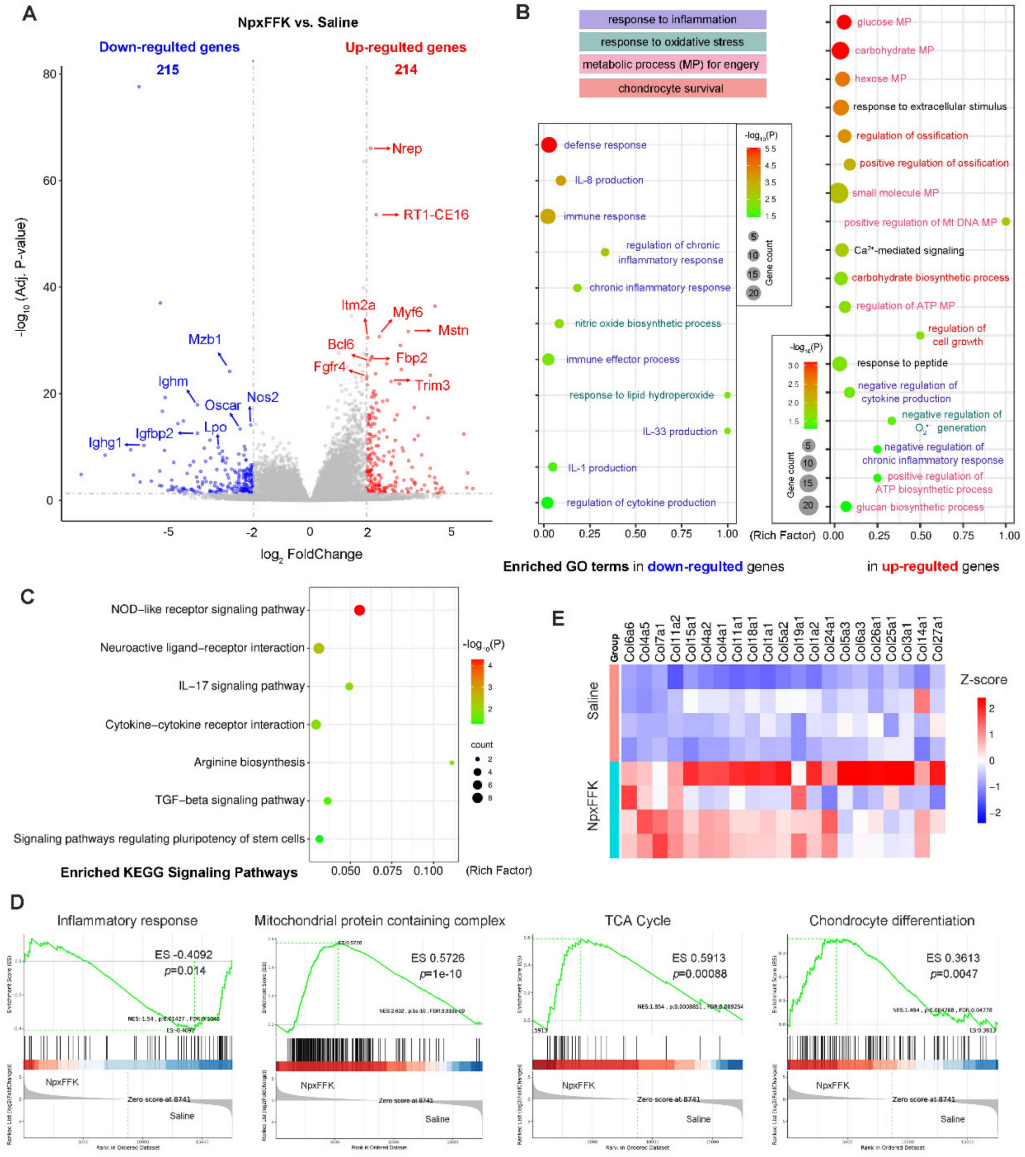
RNA sequencing analysis of the therapeutic mechanism. (**A**) Volcano plots of DEGs. (**B**) Enriched GO terms in down-regulated and up-regulated genes related to inflammatory response, oxidation response, metabolic processes (MP) and chondrocyte survival. (**C**) KEGG enrichment analysis of related signaling pathways. (**D**) GESA analysis of inflammatory response to antigenic stimulus, mitochondrial protein containing complex, citrate cycle (TCA cycle), and chondrocyte differentiation between the NpxFFK group and the saline group. (**E**) Heatmap of collagens-related genes in the saline and NpxFFK groups.

To elucidate the therapeutic mechanism of NpxFFK hydrogel in OA treatment, we conducted RNA sequencing analysis of the knee joint cartilages from the saline and NpxFFK groups (n = 4 in each group). Comparative analysis identified 429 differentially expressed genes (DEGs) (p < 0.05, log_2_(FoldChange) > 2), comprising 215 down-regulated and 214 up-regulated genes in the NpxFFK groups versus*.* saline control (Figure 9A). The most significantly down-regulated DEGs included immune response-associated genes (*Mzb1*, *Ighm*, *Ighg*), oxidant response-associated genes (nitric oxide synthase 2* Nos2* and lactoperoxidase *Lpo*), osteoclast differentiation mediator (*Oscar*), and *Igfbp2* that attenuates insulin-like growth factor (IFG) signaling in OA pathogenesis 41. Meanwhile, the most significantly up-regulated DEGs included transforming growth facter beta (TGF-β) signaling components (*Nrep* and *Mstn*), immune regulator (*RT1-CE16*), bone repair mediator (*Itm2a*) 42, involved in metabolic regulator (*Fbp2*), regulating and cell proliferation/differentiation regulators (*Myf6*, *Fgfr4* and *Trim3*). Gene ontology (GO) enrichment analysis revealed that NpxFFK hydrogel treatment significantly down-regulated inflammatory responses (defense response, immune response, chronic inflammatory response, interleukins production), and oxidation responses (NO biosynthesis, response to lipid hydroperoxide, and superoxide anion (O_2_^•-^) production) (Figure 9B). In contrast, the up-regulated genes were mainly associated with metabolic and repair processes, such as glucose metabolism, ATP metabolism and DNA metabolism, ossification, cell growth and carbohydrate biosynthesis. Kyoto encyclopedia of gene and genomes (KEGG) pathway analysis (Figure 9C) demonstrated that the DEGs were enriched in pathways related to regulation of inflammatory response (NOD-like receptor signaling pathway, IL-17 signaling pathway, cytokine-cytokine receptor interaction), oxidation response (arginine biosynthesis), cell growth and differentiation pathways (TGF-β signaling pathway and stem cells pluripotency regulating pathways), as well as pain-related pathway (neuroactive ligand-receptor interaction). Gene set enrichment analysis (GESA, Figure 9D) further indicated that NpxFFK hydrogel treatment reduced inflammatory response to antigenic stimulus, while restoring mitochondrial functions by enhancing mitochondrial protein containing complexes, particularly inner mitochondrial membrane protein complex and NADH dehydrogenase activity (Figure S22). These changes collectively activated critical energy-producing pathway including glycolysis, oxidative phosphorylation and the citrate cycle (TCA cycle), leading to increased ATP synthesis. The resulting metabolic improvements facilitated chondrocyte proliferation and differentiation (Figure 9D). This was further evidenced by upregulated expression of collagen genes (Figure 9E). Taking together, these findings established that NpxFFK hydrogel exerts its therapeutic effects through a multifaceted mechanism: reducing inflammation and oxidative stress while simultaneously enhancing cellular metabolism and energy supply to support cartilage repair and regeneration.

## Conclusion

Knee osteoarthritis, one of the most prevalent degenerative joint diseases, is characterized by progressive cartilage loss, synovial inflammation, leading to joint pain and dysfunction. While current pharmacological interventions primarily focus on managing symptoms 7,8. the limited self-repair capacity of cartilage has spurred development of regenerative approaches including surgical soft tissue coverage 43,44, biomaterial-based cartilage tissue engineering 45-47, and cell therapy 48,49. In this study, we propose a two-in-one approach for OA therapy, combining the therapeutic benefits of nonsteroidal anti-inflammatory drugs (NSAIDs) with the mechanical support and sustained delivery advantages of hydrogels. Through systemic evolution of various self-assembling peptide- naproxen conjugates, we identified NpxFFK as an efficient candidate capable of forming stable, injectable low molecular weight hydrogel (LMWH). This formulation is suitable of injectable intra-articular administration, providing sustained anti-inflammatory effects within the joint cavity. In OA rat models, NpxFFK hydrogel exhibited significant analgesic, anti-inflammatory, and chondroprotective effects. Its mechanism involved inhibiting COX-2 activity, reducing pro-inflammatory cytokine expression, promoting metabolic processes, restoring mitochondrial functions, and increasing ATP synthesis. Consequently, NpxFFK hydrogel effectively alleviated pain, improved joint function, and preserved cartilage integrity, outperforming conventional OA therapies such as naproxen and hyaluronic acid treatments. Notably, the capacity of NpxFFK hydrogel to stimulate chondrocyte proliferation/differentiation positions it as a bioactive scaffold for cartilage tissue engineering. Further development of NSAID-hydrogel systems will achieve integrative benefits in OA therapy.

## Methods

### COX-2 Inhibition Assay

Peptides were dissolved in DMSO to make a stock solution at 40 mM, and diluted into working concentrations of 400 µM in ddH_2_O. The corresponding cyclooxygenase-2 (COX*-2*) inhibition levels were detected by the Cyclooxygenase-2 Inhibitor Screening Reaction Kit (Shfksc, China) in accordance with the methodology provided by the supplier.

### Molecular Docking Calculations

Molecular docking calculations were performed with the help of AutoDock 4. The 3D structure of COX-2 was obtained in the Protein Data Bank (PDB ID: 6*COX*) and the structures of Npx and NpxFFK were optimized using Chem3D. AutoDock software files were prepared by adding polar and removing nonpolar hydrogens and binding charges to atoms. Docking of Npx or NpxFFK (ligand) was accomplished on the structure of COX-2 (receptor). Ligands were docked on the structure of COX-2 to select the energetically optimal docking configuration from the system. The analysis was performed by setting the grid size to 126, 126, and 126 on the X, Y, and Z axes, respectively (grid spacing = 0.536 Å). The docking conformations were visualized using PyMOL 2.5 from Schrodinger Software Inc.

### qRT-PCR Expression Analysis

After treatment, cells were harvested and total RNA was extracted using a total RNA purification kit (SparkJade, China), and cDNA was obtained from total RNA by a reverse transcription kit (SparkJade). Finally, qRT-PCR was performed using SYBR Green qPCR premix (SparkJade) on QuantGene 9600 real-time PCR (Bioer, Hangzhou, China). 2 Calculation of Relative Gene Expression-ΔΔCT method was used, and GAPDH was used as an internal reference gene 50. Primers were purchased from Dynaeco and Bioer, and the primer sequences used are listed in the table S2 51.

### Immunofluorescence Imaging

Immunofluorescence staining was performed following a general procedure. After treatment, cells were fixed with 4% paraformaldehyde for 20 min, and permeabilized in 0.5% Triton X-100 for 5 min. 10% goat serum was used to block non-specific binding. Cells were then incubated with primary antibodies overnight. Then cells were incubated with fluorescently labeled secondary antibody for 1 h. Subsequently, the secondary antibody was washed out, stained with DAPI for 5 min, and observed under a fluorescence microscope (Leica, Germany). For assessing RAW264.7 macrophage polarization, permeabilization procedure is not needed; anti-CD86 and anti-CD163 primary antibodies were used for identifying M1 macrophage or M2 macrophage, respectively. Primary antibodies included: anti-CD86 (1:300), anti-CD163 (1:300), anti-COX2 (1:300) and anti-COL2 (1:300). Cy3 or FITC labeled secondary antibodies were used for fluorescent imaging.

### OA Treatment

The OA model was induced by local injection of 50 µL/1mg sodium iodoacetate (MIA) into the joint cavity of each rat 52. Rats injected with saline were treated as the sham group (n = 6). After 1 week of induction time, the rats were randomly divided into 4 different groups including: (1) saline group, (2) NpxFFK group, (3) Npx group and (4) HA group for OA therapy. Each group contained 6 rats. 50 µL drugs were injected locally at day 0 and day 14, respectively. After 14 days of treatment, the rats were examined by indentation-collecting gait analysis 53, after which the rats were euthanized with isoflurane 35, and the knee joints were sampled for gross morphological observation 54. After 28 days of treatment, sampling was performed for micro-CT analysis, histological staining and immunohistochemistry.

### Gait Analysis

The cardboard is laid flat on the floor. Both forelimbs of the rats were covered with blue ink and both hind legs with red ink, ensuring that the movements of the animals were as clear as possible. The sides of the runway consisted of cardboard, and the width was rationalized according to the weight and size of the rat. The room environment was kept quiet and the rats were placed on one side of the runway and allowed to pass freely. Before the start of the experiment, the rats were given two consecutive days of behavioral acclimatization training 55.

### Histological Analysis

The knee joints were fixed in 4% paraformaldehyde, decalcified for 4 weeks, and embedded in paraffin for histological evaluation. Subsequently, the samples were sectioned and stained with hematoxylin and eosin (H&E) as well as Senna O/Fast Green. An enhanced Mankin scoring system was used to assess the pathologic status of the knee joint. Sections were incubated overnight at 4 °C with primary antibodies and further stained with a fluorescently labeled secondary antibody or 3,3'-diaminobenzidine (DAB)-tagged secondary antibody for immunohistochemical analysis.

### Micro-CT

Acquired joints were scanned with micro-CT (Bruker, SkyScan 1176, Germany) with scanning parameters of 9 mm resolution, 1 mm aluminum, 70 kV voltage and 120 mA current. 3D images were reconstructed using NRecon software. The region of interest (ROI) covered the entire subchondral bone of the tibial plateau. Three-dimensional structural parameters analyzed included bone volume/total volume (BV/TV), trabecular number (Tb.N), and trabecular separation (Tb.S).

### RNA Sequencing

RNA sequencing was performed on knee cartilages from OA rats that were treated with saline and NpxFFK hydrogel for 4 weeks (n = 4). After treatment, the rats were euthanized with isoflurane, and the knee joints were collected. Excess muscle tissues were removed, and the knee cartilages were quickly transferred to liquid nitrogen for rapid freezing for half an hour. Total RNA was harvested using TRIzol reagent (Invitrogen, Carlsbad, CA). RNA sequencing was performed by Orbio Technology Ltd (Shanghai, China).

### Statistical Analysis

All values are expressed as mean ± standard deviation. Significance analysis was assessed using one-way analysis of variance (ANOVA, Tukey post hoc test). Differences were statistically significant when p < 0.05. Statistical significance is shown as *p < 0.05, **p < 0.01, ***p < 0.001, ****p < 0.0001.

## Supplementary Material

Supplementary materials and methods, figures and tables.
