# Supplementary material for: Self-Assembling Hydrogels of Naproxen-Conjugated Peptides for Osteoarthritis Treatment
Source: Theranostics. 2025 Aug 11;15(17):8779–94. doi: 10.7150/thno.114781 (PMC12439141; doi:10.7150/thno.114781)
Supplement: Supplementary file 1 — Supplementary materials and methods, figures and tables. [file thnov15p8779s1.pdf]

Supporting Information

Self-assembling hydrogels of naproxen-conjugated peptides for osteoarthritis treatment

Lulu yang [1,2,3, #], Liang Shao [1,2, #], Puhua Hao [1,3], Jiaqi Song [2], Caiting Meng [2], Bin Zhu [2], Hongwen Yu [1,2], Wanglin Duan [2], Xiaohua Fang [2,3], Guanying Li [2,*], Shichang Liu [1, 3, *]

[1] Department of Spine Surgery, Honghui Hospital, Xi'an Jiaotong University, Xi'an, Shaanxi, 710054, P. R. China;

[2] Department of Biophysics, School of Basic Medical Sciences, Health Science Centre, Xi'an Jiaotong University, Xi'an, Shaanxi, 710061, P. R. China.

[3] The Second Clinical Medical School, Shaanxi University of Chinese Medicine, Xianyang, Shaanxi, 712046, P. R. China.

# These authors contribute equally to this work.

* *Corresponding authors: guanyingli@xjtu.edu.cn; lsc_2002@outlook.com.*

Materials and instruments

Amino acids and resin used in the solid-phase peptide synthesis were purchased form GL Biochem (Shanghai) Ltd. China. Naproxen and sodium hyaluronate were purchased from Macklin Co. The mouse monocyte macrophage cell line (Raw264.7) and human chondrocyte cell line (C28/I2) were purchased from the American Typical Culture Collection (ATCC), and Dulbecco's modified Eagle's medium (DMEM), fetal bovine serum (FBS) and penicillin-streptomycin Amphotericin Liquid (100X) were purchased from Gibco (Waltham, Massachusetts). Calcein-AM/PI double staining kit for living and dead cells was purchased from Elab Science (China). ROS detection kit was purchased from Biosharr (China), and MTT was purchased from Solarbio (China). Cyclooxygenase-2 inhibitor screening kit was purchased from Beyotime (China). Primary antibodies included: anti-CD86 was purchased form Bioss, China; anti-CD136, anti-COX-2 and anti-COL2 were purchased form Affinity Biosciences, China. Secondary antibodies were purchased from Affinity Biosciences (China). ELISA kits were purchased from Shfksc, China. SPARK easy Cell RNA Kit, SPARK script" RT Plus kit, and SYBR Green qPCR Master Mix were purchased from Spark Jade Biotech Co.

The naproxen-peptide conjugates were purified on an EClassical 3140 HPLC (Elite, China), and their mass spectra were measured with a mass spectrometer Agilent-6125B ESI (Agilent, Santa Clara, CA, USA). The ^{1}H NMR spectra were recorded on a 400 MHz JEOL spectrometer (JEOL, Tokyo, Japan). The chemical shifts (δ) were given in ppm referred to the internal standard tetramethylsilane (TMS). Rheological data were collected using a HAAKE MARS rheometer (Thermo) with an environmental test chamber. TEM images were obtained with a Talos L120C transmission electron microscope. FT-IR spectra were recorded in attenuated total reflection (ATR) mode using a Bruker Vertex 70 FTIR spectrophotometer and analyzed by OriginPro 2021 software (student edition, copyright® Originlab, USA). Fluorescence images were captured on a DMi8 fluorescence inverted microscope (Leica, Wetzlar,

Germany). Zeta potential measurements were performed on an Anton Paar Litesizer 500 (Austria).

Synthesis

Peptides NpxFFX (X = H/R/E/K) were synthesized by solid-phase peptide synthesis (SPPS) using 2-chlorotriphenyl chloride resin and Fmoc-protected amino acids (Scheme S1). The crude products were purified on an EClassical P3140 HPLC system (Elite, China) equipped with a SinoChrom C18 chromatographic column (ODS-BP, 10 μm, 20.0 mm X 250 mm), obtaining white powders.

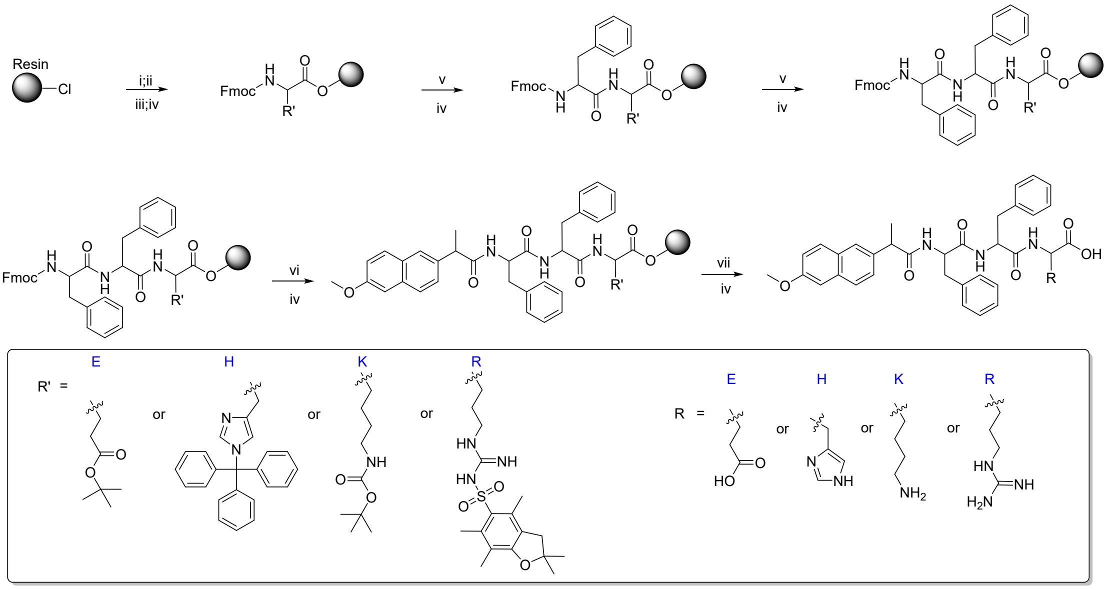

Scheme S1. Synthesis of peptide NpxFFE, NpxFFH, NpxFFK and NpxFFR. Reagents and conditions: (i) DCM; (ii) Fmoc-Glu(tBu)-OH or Fmoc-His(Trt)-OH or Fmoc-Lys(Boc)-OH or Fmoc-Arg(Pbf)-OH; DIEA, DMF; (iii) DIEA: MeOH: DCM (5:15:80); (iv)5%Piperazine, 2%DBU, DMF; (v)Fmoc-Phe -OH, HBTU, DIEA, DMF; (vi) Npx, HBTU, DIEA, DMF; (vii) 95%TFA, 5%DCM.

NpxFFE: Yield 46.6%. ESI-MS (m/z) calcd. for $C_{37}H_{39}N_3O_8$, 653.2737; found $[M+H]^+$ 654.2023, and $[M+Na]^+$ 676.1819. ^1H NMR (400 MHz, DMSO-*d6*) δ 8.19 – 7.99 (m, 3H), 7.77 – 7.56 (m, 3H), 7.40 – 7.00 (m, 13H), 4.50 (ddd, *J* = 19.9, 11.8, 5.6 Hz, 2H), 4.19 – 4.13 (m, 1H), 3.86 (S, 3H), 3.77 – 3.66 (m, 1H),

3.09 – 2.91 (m, 2H), 2.80 – 2.63 (m, 2H), 2.36 – 2.17 (m, 2H), 1.95 – 1.71 (m, 2H), 1.25 (dd, *J* = 55.4, 7.0 Hz, 3H).

NpxFFH: Yield 50.3%. ESI-MS (m/z) calcd. for $C_{38}H_{39}N_5O_6$, 661.2900; found $[M+H]^+$ 662.2216. ^{1}H NMR (400 MHz, DMSO-*d6*) δ 8.28 (d, J = 7.5 Hz, 1H), 8.10 (dd, J = 24.8, 8.4 Hz, ^{2}H), 7.79 – 7.47 (m, 4H), 7.37 – 6.76 (m, 12H), 4.67 – 4.34 (m, 3H), 3.85 (d, J = 6.9 Hz, 3H), 3.71 (d, J = 7.2 Hz, 1H), 3.12 – 2.82 (m, 4H), 2.76 – 2.60 (m, 2H), 1.25 (dd, J = 55.3, 7.1 Hz, 3H).

NpxFFK: Yield 56.1%. ESI-MS (m/z) calcd. for $C_{48}H_{44}N_4O_6$, 652.3261; found $[M+H]^+$ 653.2576. ^{1}H NMR (400 MHz, DMSO-*d6*) δ 8.19 – 7.99 (m, 3H), 7.77 – 7.56 (m, 4H), 7.40 – 7.00 (m, 12H), 4.50 (ddd, J = 19.9, 11.8, 5.6 Hz, 2H), 4.19 – 4.13 (m, 1H), 3.90 – 3.81 (m, 3H), 3.77 – 3.66 (m, 1H), 3.09 – 2.91 (m, 2H), 2.80 – 2.63 (m, 4H), 2.36 – 2.17 (m, 1H), 1.95 – 1.71 (m, 3H), 1.25 (dd, J = 55.4, 7.0 Hz, 3H).

NpxFFR: Yield 55.7%. ESI-MS (m/z) calcd. for $C_{38}H_{44}N_6O_6$, 680.3322; found $[M+H]^+$ 681.2581. ^{1}H NMR (400 MHz, DMSO-*d6*) δ 8.25 (dd, J = 47.7, 8.6 Hz, 2H), 7.84 – 7.47 (m, 4H), 7.39 – 6.74 (m, 12H), 4.68 – 4.26 (m, 2H), 3.98 – 3.81 (m, 3H), 3.72 (dq, J = 14.0, 6.9 Hz, 1H), 3.14 – 2.86 (m, 3H), 2.80 – 2.57 (m, 2H), 1.62 (s, 2H), 1.42 (t, J = 26.4 Hz, 2H), 1.35 – 1.10 (m, 3H).

Fourier Transform Infrared (FT-IR) Spectra

500 μL of peptide NpxFFX (X = H/R/E/K) samples at 400 μM in ddH_2O were used for the assay. The recorded wave number range was 4000-400 cm^{-1}.

Rheological analysis

25 mg/mL peptide was prepared in PBS at room temperature and placed in printed molds with a diameter of 25 mm and a thickness of 1 mm using a parallel plate geometry, and the samples were placed between a stainless steel upper and lower Peltier plate with a diameter of 20 mm, with a gap of 1.0 mm between all measurement plates. Dynamic strain sweeps were performed in the range of 0.01-100%, and slope logarithmic profiles were performed on the data points. The self-healing properties with amplitude oscillatory strain was switched from small strain (γ = 0.1% at 100 s intervals) to large strain (γ =

100% at 100 s intervals) for six cycles. The storage modulus (G') and loss modulus (G") were plotted on a logarithmic scale with dynamic strain sweep and amplitude oscillatory strain scans, respectively.

TEM Imaging

A drop of 10 μL peptide samples was added to a discharge copper grid coated with a carbon film and left for 10 minutes. Afterwards, the excess solution was removed by pipetting with filter paper, the grid was washed with pure water and stained with 1% uranyl acetate for 60 seconds. The grids were allowed to dry at room temperature and prepared for TEM imaging.

Zeta potentials measurement

Samples (400 μM) were prepared in phosphate buffered solution (10 mM pH 7.2). Their zeta potentials were recorded by laser diffraction particle size analyzer (Anton Paar Litesizer 500) at room temperature.

In vitro Drug release degradation test

500 μL 2 mg/mL NpxFFK (containing 2 mg/L, 1mg/L, 0.5 mg/L, 0.25 mg/L, 0.125mg/L,0.0625 mg/L) gel was loaded into a 3D printed mould and soaked in 2.5mL ddH2O at 37°C. At predetermined intervals, 100 μL medium was collected to measure absorbance, and 100μL fresh ddH2O was supplemented to keep the total volume constant. The cumulative release of NpxFFK was quantified by reference to the calibration curve of ultraviolet absorption of sodium flavin phosphate. The NpxFFK release rate is calculated as a percentage of the NpxFFK released to the total NpxFFK load.

2 mL 25 mg/mL NpxFFK was loaded into a dialysis bag and placed in 48 mL of ddH2O at 37 ^{o}C. At predetermined time intervals, 100 μL of the release medium was collected for ultraviolet absorption analysis, and 100 μL of fresh ddH2O was replenished to maintain a constant total volume. The cumulative release of NpxFFK was quantified based on their ultraviolet absorption peak areas and corresponding concentration calibration curves. The release rate (%) of NpxFFK was calculated as the percentage of released NpxFFK relative to their total initial loading amount.

DPPH free radical scavenging test

DPPH (2, 2-Diphenyl -1-trinitrohydrazyl) radical solution (0.58 mg/mL) was prepared by dissolving DPPH radical in anhydrous ethanol. Briefly, 6mg of NpxFFX (X=H/R/E/K) samples and Npx were added to 3 mL of DPPH radical solution which were then incubated in dark for 2 h at 25°C. The absorbance of the mixed solution at 517 nm was measured using an enzyme-labeled instrument. The scavenging ability was calculated as DPPH free radical scavenging rate (%) = [(A0-A1)/A0] × 100%, where A0 is the absorbance of DPPH free radical solution, A1 is the absorbance of sample group. The experiment was repeated three times for each sample.

ABTS·+ free radical scavenging test

25 mL 2,2'-azino-bis(3-ethylbenzothiazolin-6-sulfonic acid) radical solution (0.8 mg/mL) was mixed with 25 mL potassium persulfate solution (0.14 mg/mL) evenly to form $ABTS^+$ free radical solution, and then incubated in dark for 2 h. NpxFFX (X=H/R/E/K) samples and Npx were added to 3 mL of $ABTS^+$ free radical solution, and then incubated at 25°C for 30 mins in dark. The absorbance at 734 nm was measured using an enzyme-labeled instrument. $ABTS^+$ free radical scavenging activity is calculated according to the following formula: $ABTS^+$ free radical scavenging rate (%) = [(A0-A1)/A0] × 100%, where A0 is the absorbance of $ABTS^+$ free radical solution, and A1 is the absorbance of sample group. The experiment was repeated three times for each sample.

Cell Culture

Mouse primary macrophage RAW264.7, human chondrocyte C28/I2 are cultured in Dulbecco's modified Eagle's medium (DMEM) containing 10% fetal bovine serum (FBS) and antibiotics. All cells were maintained at 37 ℃ and 5% CO_2.The medium was changed every 24 h.

MTT assay

C28/I2 cells were inoculated in 96-well plates at a density of 5000 cells per

well. After cells adhesion, medium was replaced with DMEM containing 125, 250, 500, 1000, and 2000 μM samples (n = 3). The cells were then incubated in an incubator for 24 h. Subsequently, DMEM medium containing MTT was added and incubated for 4 hours in the dark. The medium was then removed and the precipitates was dissolved with DMSO. Finally, the optical density (OD) at 570 nm was measured using a Bio-Tex (Elx 800) enzyme marker.

Live/dead staining assay

C28/I2 cells were cultured in 48-well plates (Corning, USA) and 400 μM peptides solutions were added to the supernatant for co-incubation. On day 1, 2 and 3, the cells were washed with fresh media and stained with Calcein AM/PI assay solution (elabscience) at 37℃ for 30 min. After washed three times with PBS, cells were imaged under a fluorescence microscope.

Hemolysis test

The bloods were collected from SD rats. The blood was mixed with saline and centrifuged until the supernatant is colorless and clear. Red blood cells were obtained and mixed with saline to form a 2% cell suspension. Peptide samples were prepared at a concentration of 500 μg/ml in saline. Then, 0.5 mL of diluted red blood cell suspension was added to each sample (0.5 mL) and the mixture was incubated at 37 °C for 1 hour. ddH_2O (0.5 mL) was used as a positive control and saline as a negative control. All samples were centrifuged at 3000 rpm for 5 minutes and the supernatant was collected. The OD of the supernatant was measured using a microplate analyzer at 450 nm. the percentage of hemolysis was calculated using the following formula:

Hemolysis Rate(%) = $(OD_{sample} – OD_{saline}) / (OD_{water} - OD_{saline})$ x 100%.

Mitochondrial membrane potential detection

After incubating chondrocytes C28/I2 for 12h using a six-well plate, the culture media of RAW 264.7 after stimulation with LPS (1.0 μg/mL) for 6 h were collected and transferred to the C28/I2 cell culture. C28/I2 cells were treated with NpxFFK, Npx, and HA for another 24 h and the changes in mitochondrial membrane potential were detected by JC-1 staining. Cells were incubated

with JC-1 dye for 30 min at 37 ℃ in an incubator, then washed thoroughly and the results were visualized using an inverted fluorescence microscope (Leica, Germany).

Fluorescence detection of ROS

RAW 264.7 cells were co-incubated with LPS (1.0 μg/mL) and 400 μM of NpxFFK, Npx, and HA for 12 h. Then, the cells were incubated with DCFH-DA (10 μM) in an incubator at 37 ℃ for 20 min and washed with PBS for 3 times. Intracellular ROS were observed using an inverted fluorescence microscope (Leica, Germany). The fluorescence intensity was measured using an multiplate reader (BioTek SynergyTM 4) for quantitative statistics.

In vivo imaging

8-week-old female SD rats were randomly divided into three groups. 1 mg NIR dye IR783 was dissolved in 100 μL saline and thoroughly mixed with 100 μL NpxFFK, Npx or HA at 20 mg/ml concentration. 50 μL mixture was injected into the joint cavity of rats. In vivo fluorescence images were captured at 0, 3, 7, and 14 days using the VISQUE in vivo Smart-LF imaging system.

Magnetic Resonance Imaging (MRI)

Acquired joints were fixed in the MRI detector with a 23 mm surface coil for the scanning coil, and examined by a Time Medical 7.0 T small animal MRI machine (NOVA 7T/160, Preclinical Magnetic Resonance Imaging System, Time Medical, USA), employing T2-weighted sequences based on fast echo sequences. T2-weighted images were acquired employing the sequence parameters: TR: 3000 ms; TE: 17.26 ms; FOV: 20 × 20 (mm); Average: 2; Data matrix: 256 × 256; Slices: 15; Thickness: 0.5 mm.

Supplementary Tables

Table S1 Binding energies and Ki of COX-2 binding to Npx or NpxFFK

COX-2 inhibitor	Binding Energy	Ki
Npx	-9.36 kcal/mol	136.7 nM
NpxFFK	-9.06 kcal/mol	227.9 nM

Table S2 Sequences of Primers used for RT-qPCR

**Gene**	**Primer Sequence (5'-3')**
H-MMP13	F: AGACCTCCAGTTTGCAGAGC
	R: TACGGTTGGGAAGTTCTGGC
H-COL2A1	F: GTAGAGACCCGGACCCGC
	R: ACTCTCCGAAGGGGATCTCA
H-β-actin	F: ACAGAGCCTCGCCTTTGC
	R: GCGGCGATATCATCATCC
H-GAPDH	F: CTAAAGGGCATCCTGGGC
	R: TTACTCCTTGGAGGCCAT
M-IL-1β	F: TGCCACCTTTTGACAGTGATG
	R: TGATGTGCTGCTGCGAGATT
M-COX-2	F: TTCAACACACTCTATCACTGGC
	R: AGAAGCGTTTGCGGTACTCAT
M-GAPDH	F: TCAGGAGAGTGTTTCCTCGT
	R: ATGAAGGGGTCGTTGATGGC
M-Actin	F: GATGGTGGGAATGGGTCAGAA
	R: ATTGTAGAAGGTGTGGTGCCA
M-IL-6	F: AGAAATGATGGATGCTACCAAACT
	R: TTGGATGGTCTTGGTCCTTAGC
M-TNF-a	F: TTCCAGAACTCCAGGCGGTG
	R: CACTTGGTGGTTTGCTACGACG
M-IL-1	F: CCCAAGCAATACCCAAAGAAGAAG
	R: TGTCCTGACCACTGTTGTTTCC

H: human source; M: mouse source.

Supplementary Figures

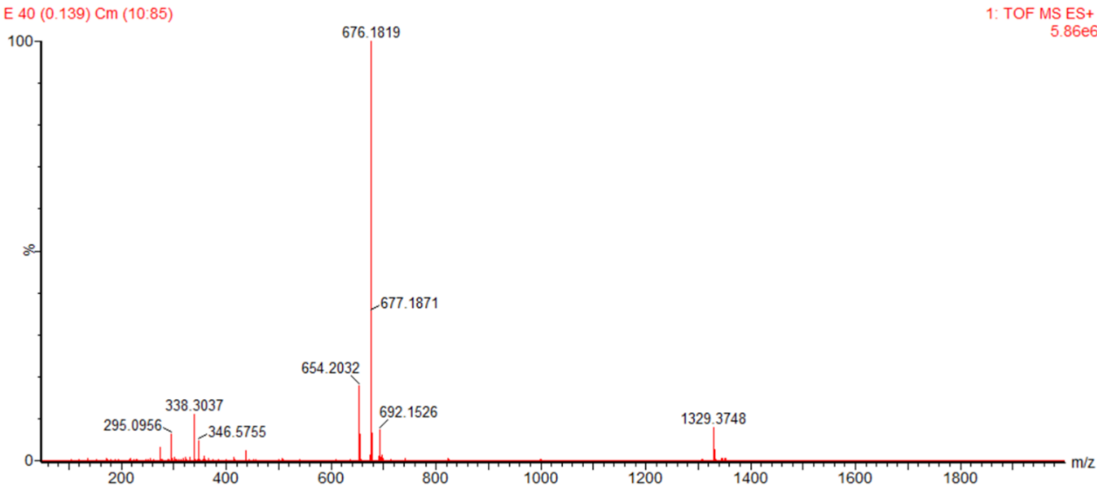

Figure S1. High-resolution mass spectrum of NpxFFE.

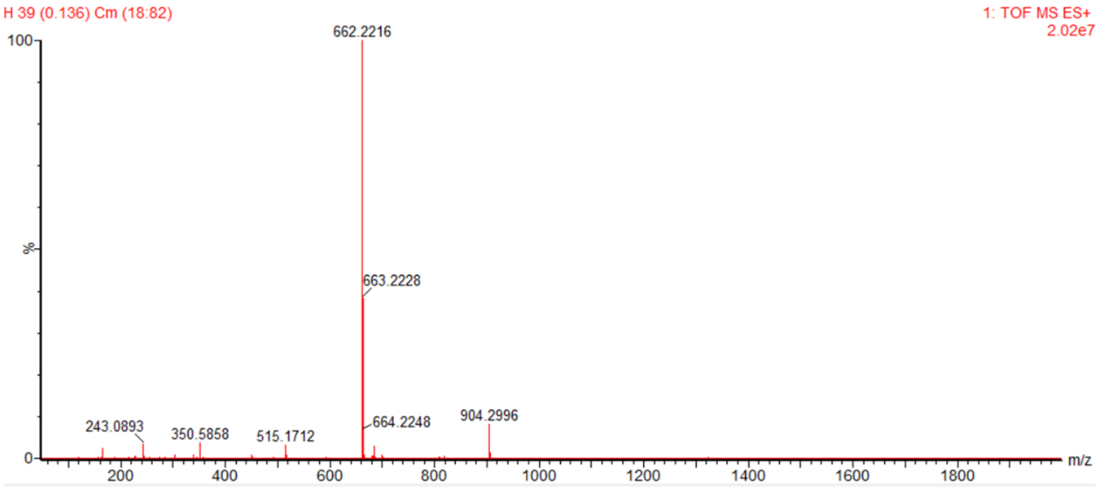

Figure S2. High-resolution mass spectrum of NpxFFH.

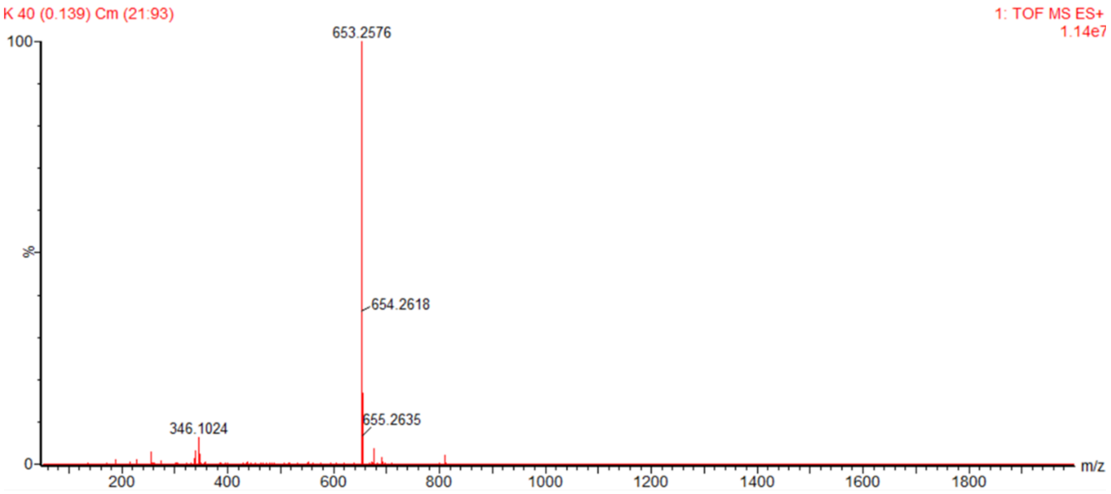

Figure S3. High-resolution mass spectrum of NpxFFK.

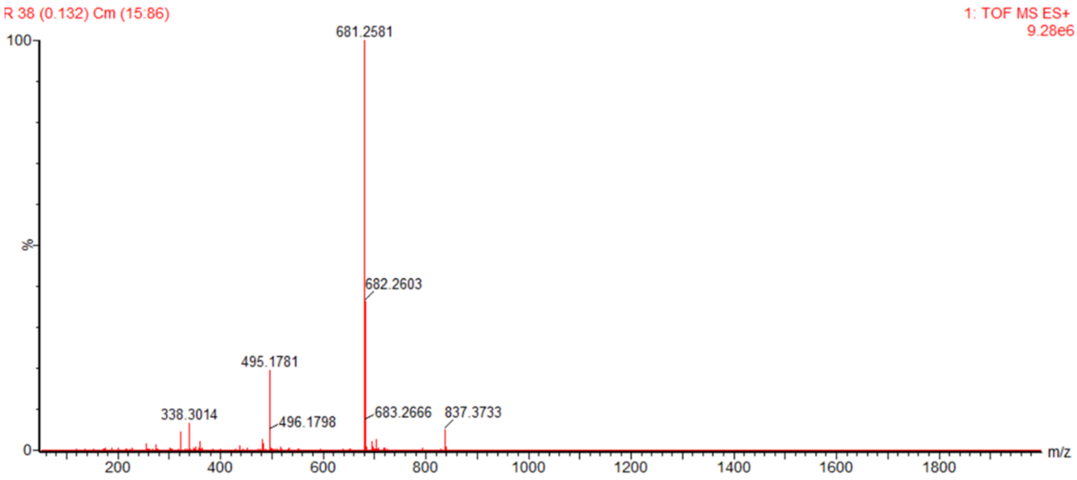

Figure S4. High-resolution mass spectrum of NpxFFR.

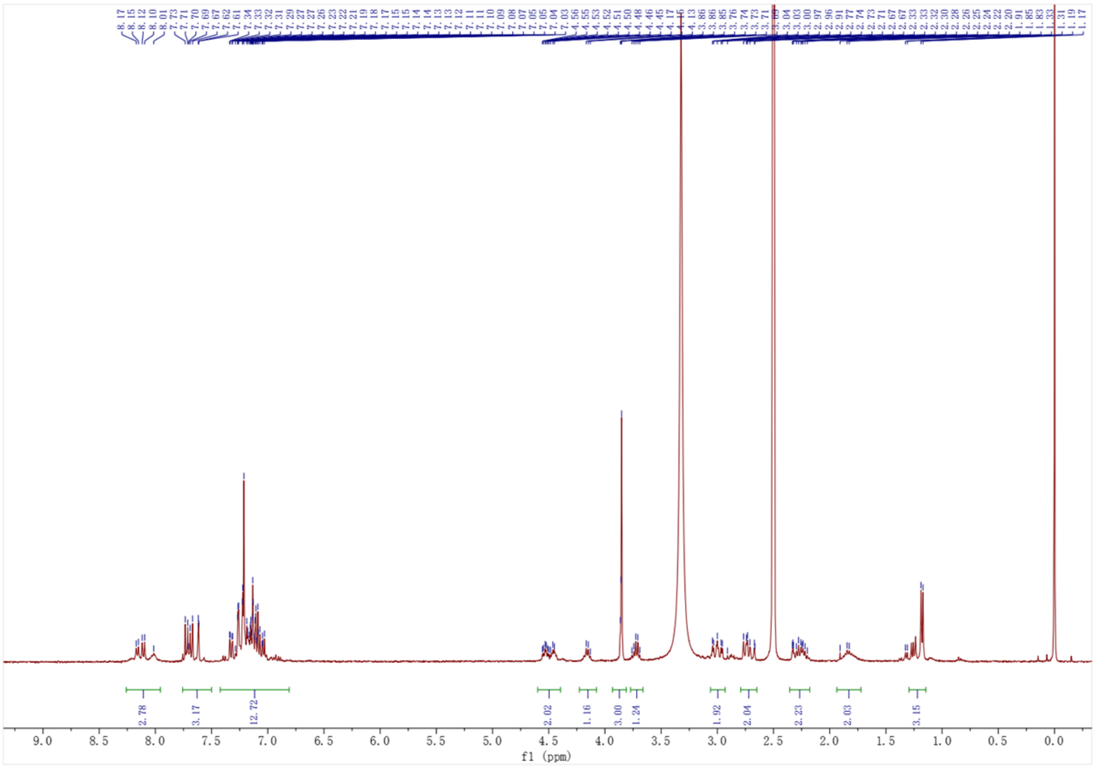

Figure S5. ^{1}H NMR spectrum of NpxFFE in DMSO-*d6*, 400MHz..

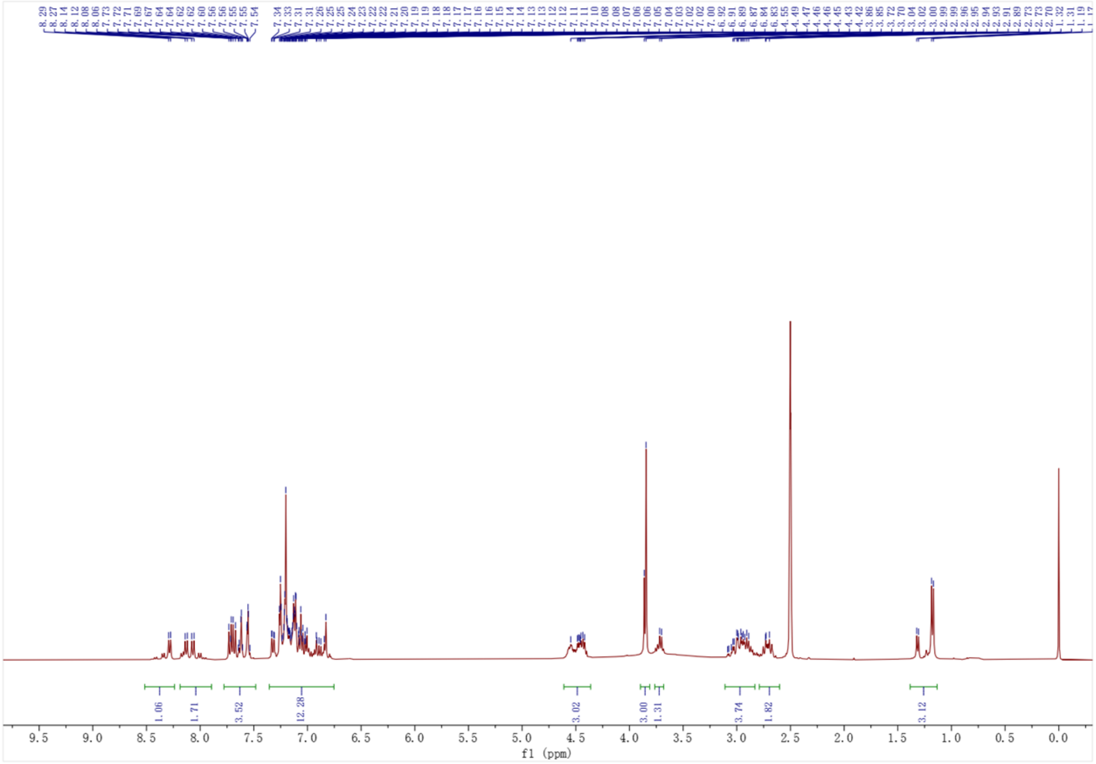

Figure S6. ^{1}H NMR spectrum of NpxFFH in DMSO-*d6*, 400MHz.

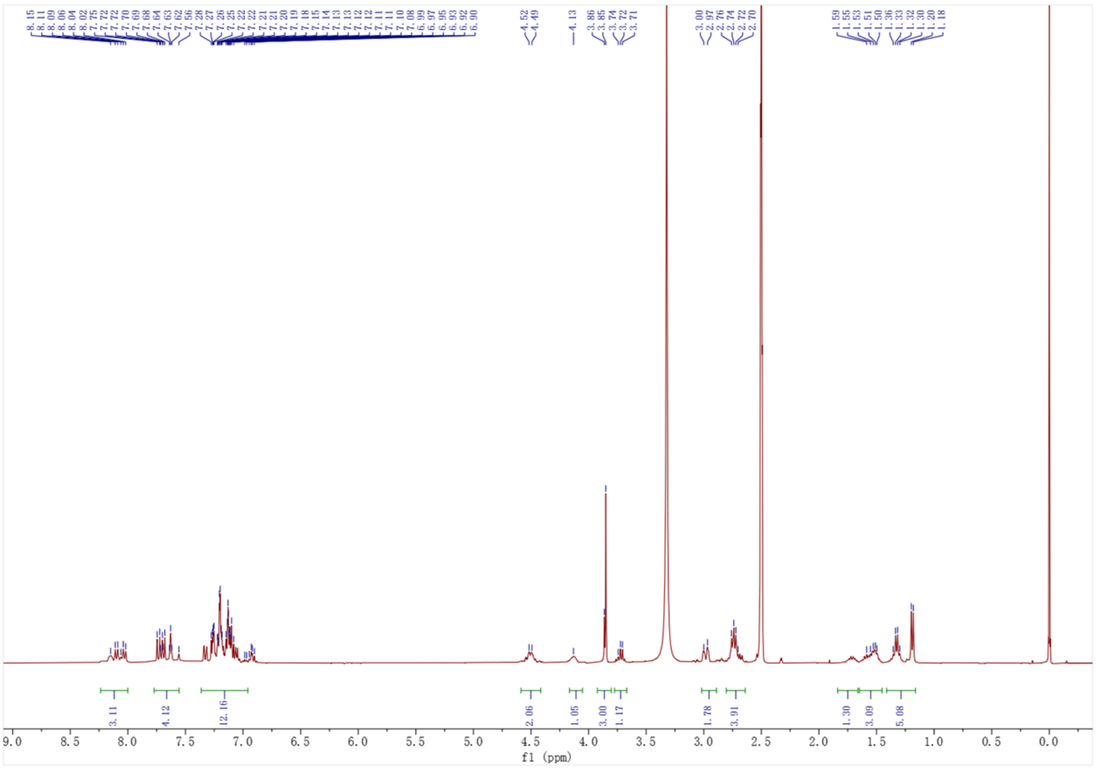

Figure S7. ^{1}H NMR spectrum of NpxFFK in DMSO-*d6*, 400MHz.

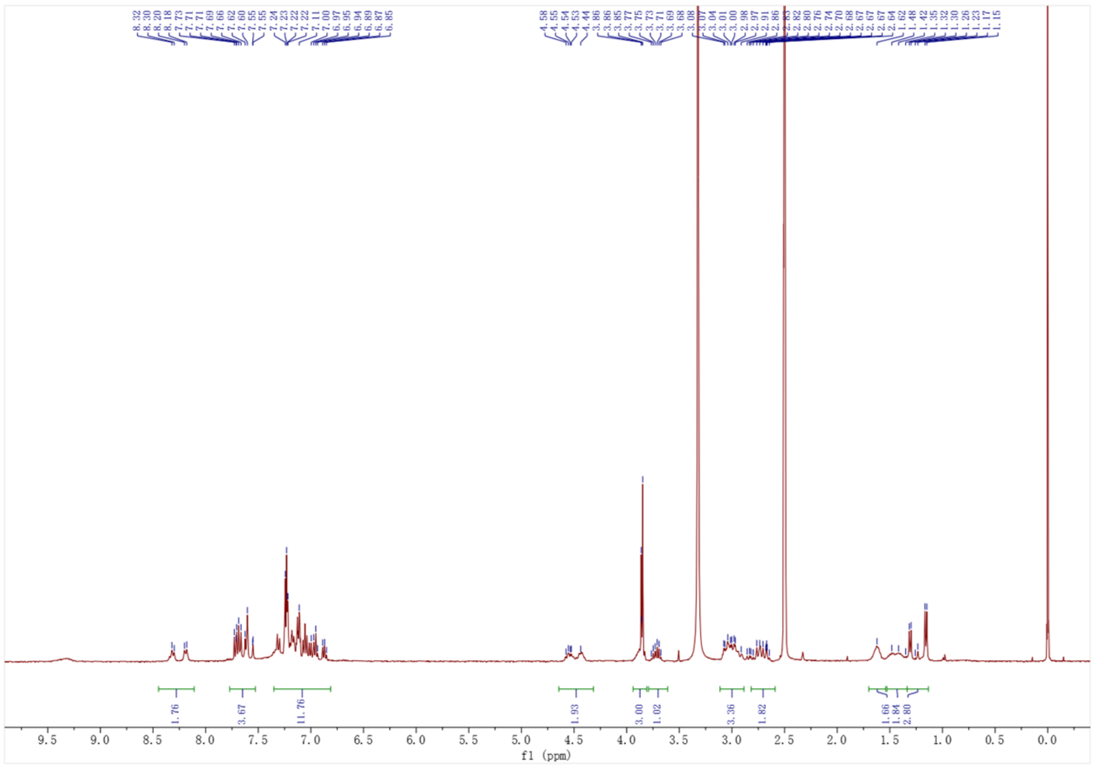

Figure S8. ^{1}H NMR spectrum of NpxFFR in DMSO-*d6*, 400MHz.

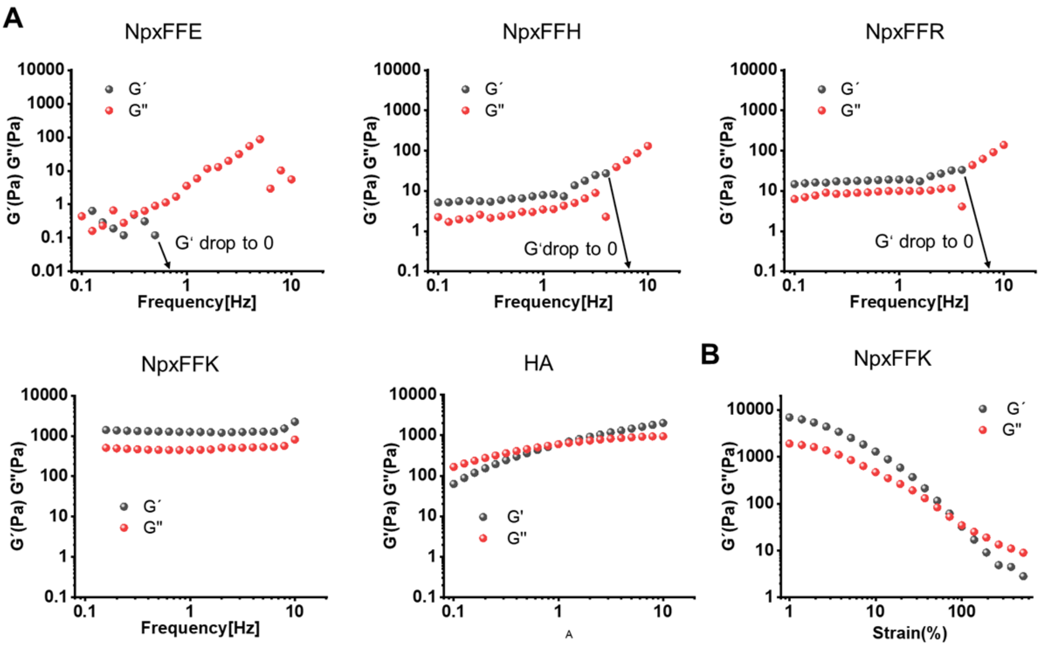

Figure S9. (A) Frequency sweep rheological tests of naproxen-peptide conjugates NpxFFE, NpxFFH, NpxFFR, NpxFFK, and HA in PBS, pH 7.2. (B) Strain sweep rheological tests of NpxFFK hydrogel in PBS, pH 7.2.

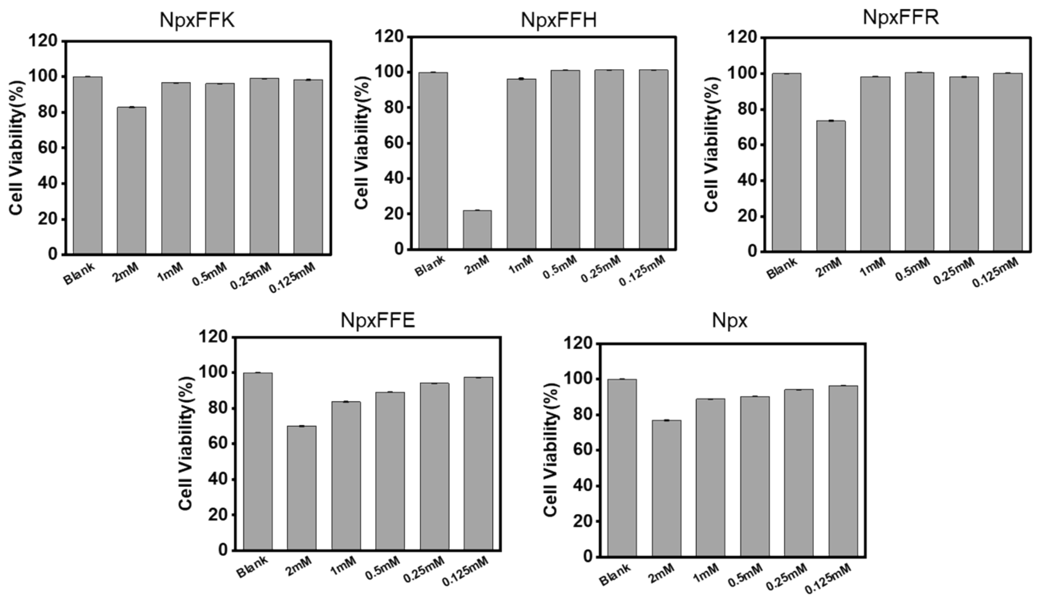

Figure S10. C28/I2 chondrocytes were treated with different concentrations (2.0, 1.0, 0.5, 0.25, or 0.125 mM) of NpxFFK, NpxFFH, NpxFFR, NpxFFE or Npx for 24 h and the cell viabilities were assessed by MTT assay. The blank groups referred to the cells treated with growing media.

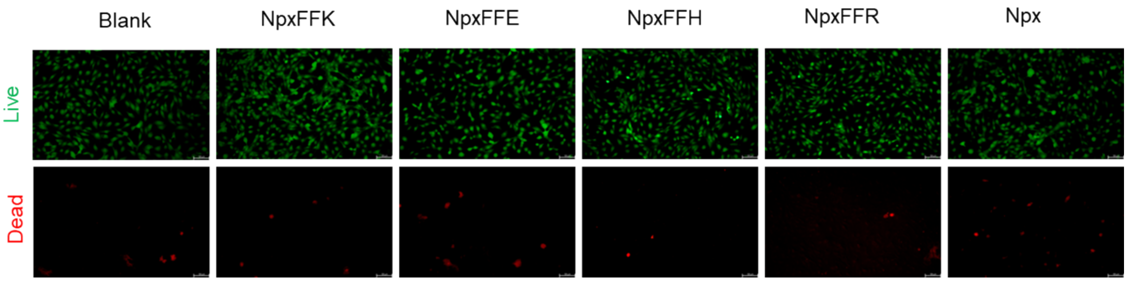

Figure S11. C28/I2 cells were treated with 400 μM of NpxFFK, NpxFFH, NpxFFR, NpxFFE or Npx for 48 h and further stained with Calcein-AM/PI staining. Green represents living cells; Red represents dead cells. Scale bar =100 μm. The blank groups referred to the cells treated with growing media.

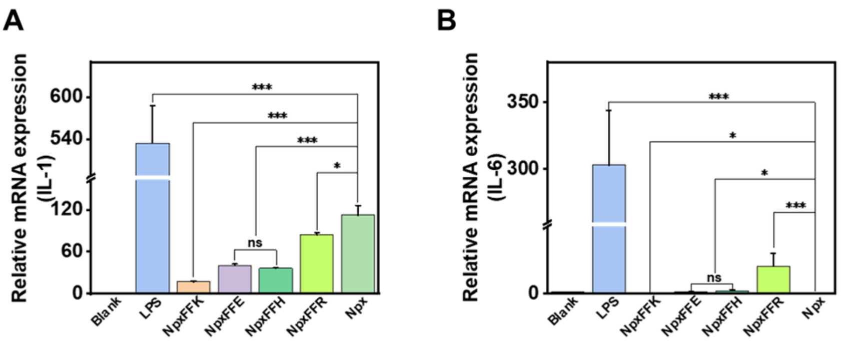

Figure S12. Relative gene expression level of IL-1 and IL-6 on LPS-stimulated RAW 264.7 cells after treatment with peptides (NpxFFH, NpxFFR, NpxFFE, NpxFFK) or Npx for 12h. n = 3, ****p < 0.0001, ***p < 0.001, **p < 0.01. The blank groups referred to the cells without stimulation of LPS.

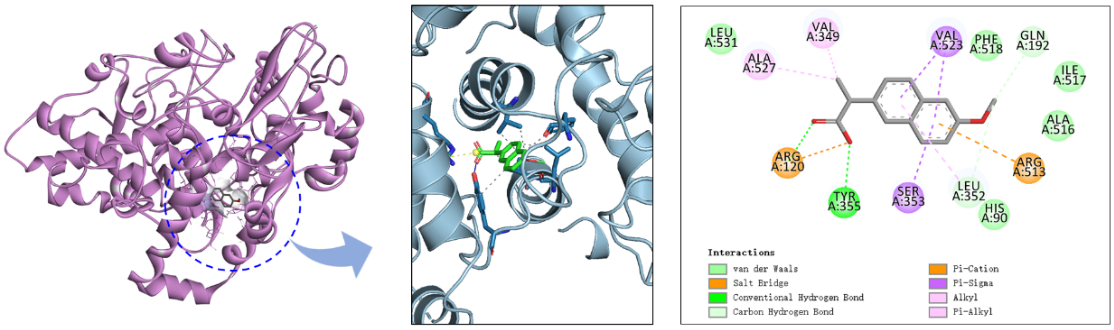

Figure S13. Molecular docking of COX-2 (PDB ID: *6COX*) with Npx.

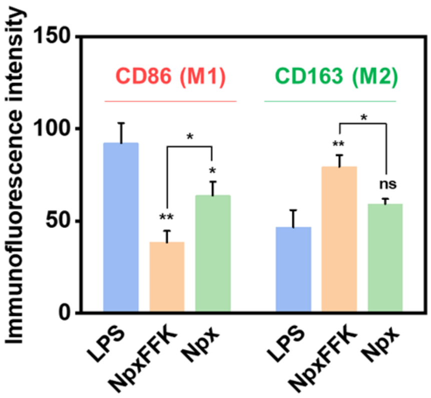

Figure S14. Quantitative analysis of immunofluorescent staining of macrophages in Figure **5A** and **5B**.

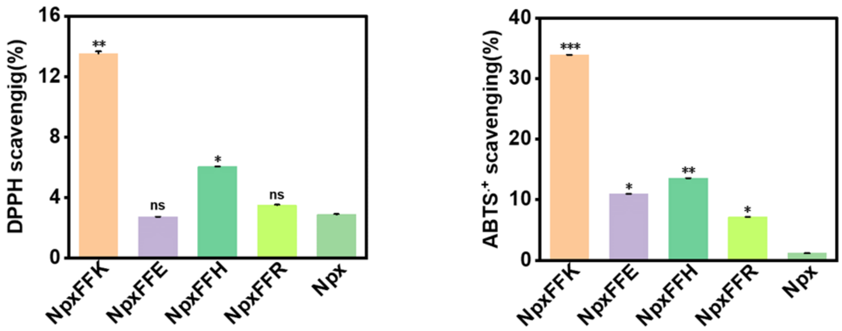

Figure S15. DPPH scavenging activities and $ABTS^+$ scavenging activities of NpxFFK, NpxFFH, NpxFFR, NpxFFE and Npx in PBS (pH 7.2) at 400 μM. n = 3, mean ± SD.

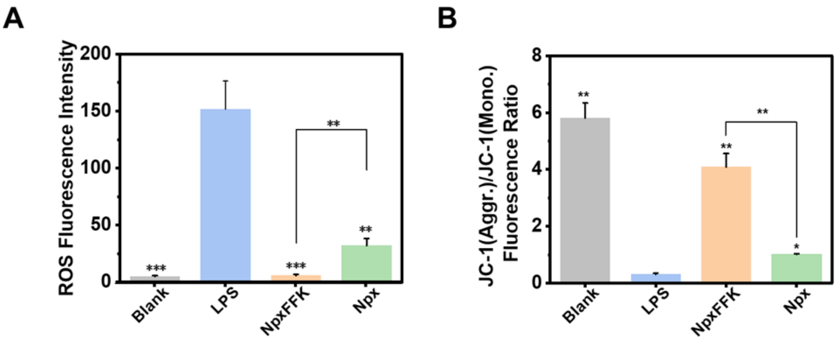

Figure S16. Quantitative analysis of ROS fluorescent intensities (**A**) in Figure **5D**, and fluorescent ratio of JC-1 in aggregate to monomer (**B**) in Figure **5E**.

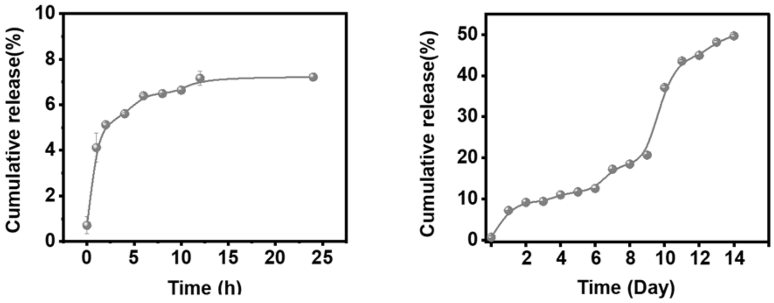

Figure S17. Degradation profiles of NpxFFK hydrogel in PBS (pH 7.2) at 20 mg/mL for 0-24 hours and 1-14 days.

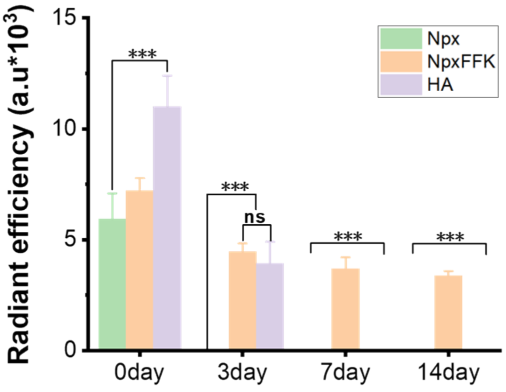

Figure S18. Quantitative analysis of *in vivo* fluorescence intensity of IR783 loaded with NpxFFK hydrogel, Naproxen solution or HA hydrogel for 0, 3, 7 and 14 days.

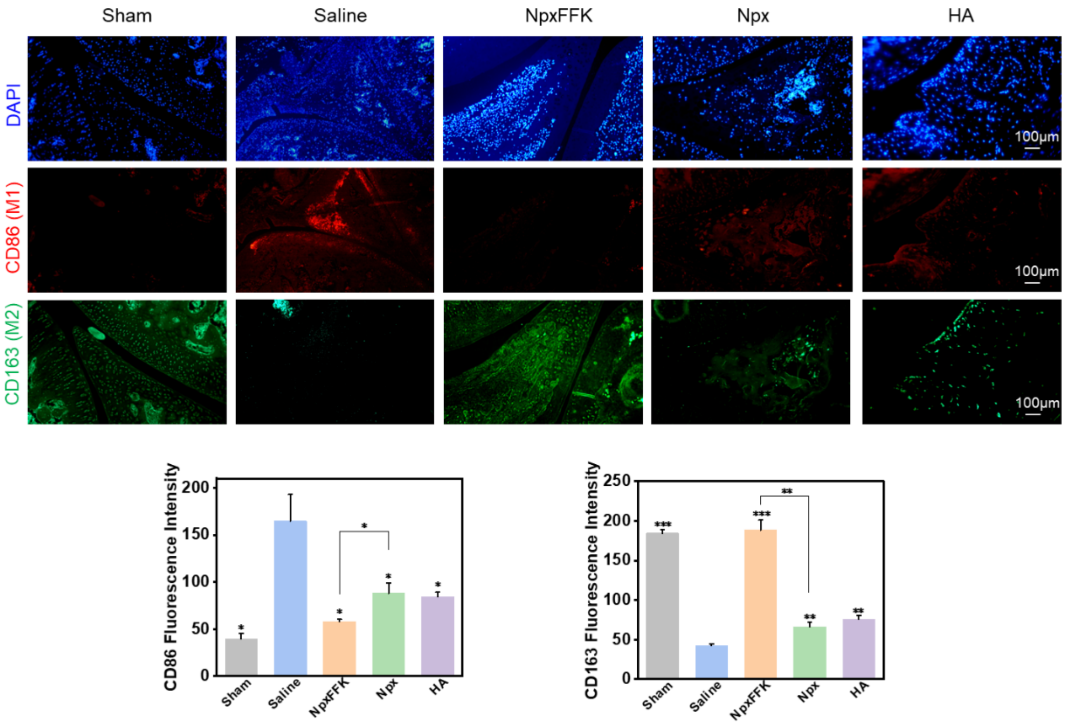

Figure S19. Representative immunofluorescence staining merged images of CD86 (M1 marker) and CD163 (M2 marker) from the sham, saline, NpxFFK, and HA groups. The scale bar represents 100 μm. Quantitative analysis of immunofluorescence intensities of M1 macrophage and M2 macrophage are also presented.

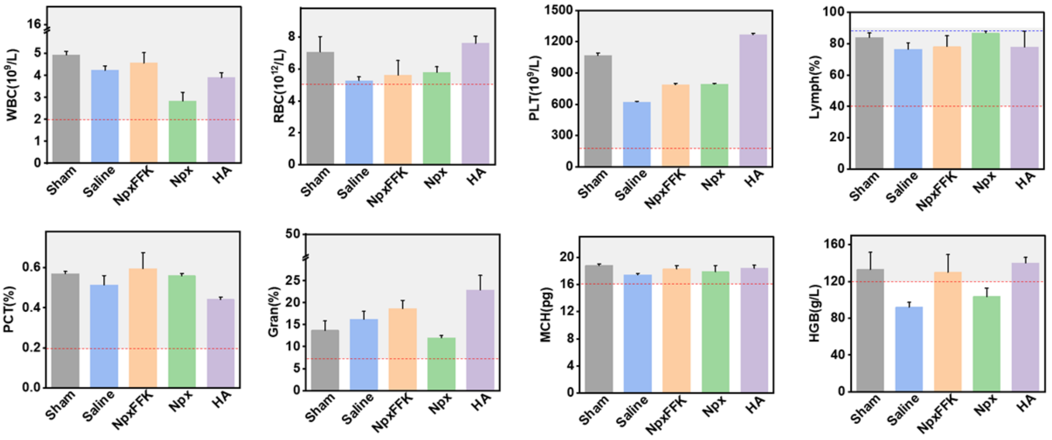

Figure S20. Quantitative statistics of blood-related indices in SD rats, including WBC, MCH, RBC, HGB, PLT, PCT, Gran and Lymph.

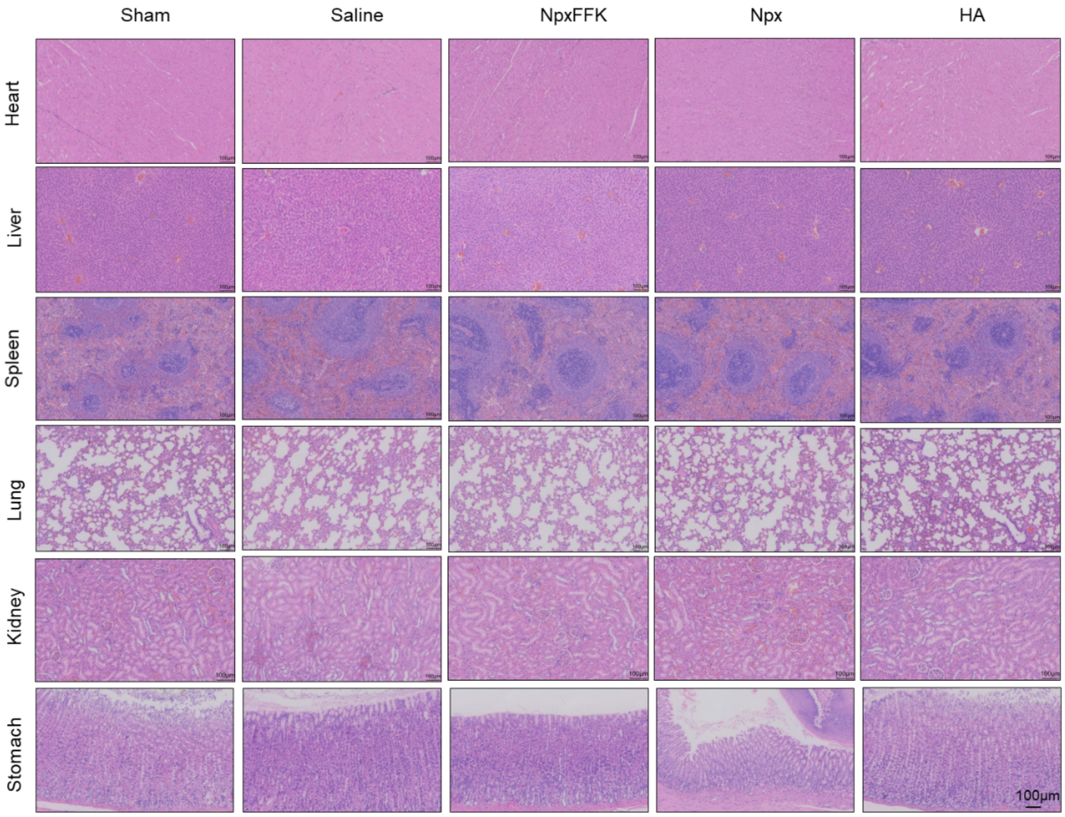

Figure S21. Representative H&E staining images of heart, liver, spleen, lung, kidney, and stomach sections separated from SD rats after treatment with saline, or with 100 mg/kg Npx, NpxFFK or HA for 4 weeks. Scale bars represent 100 μm.

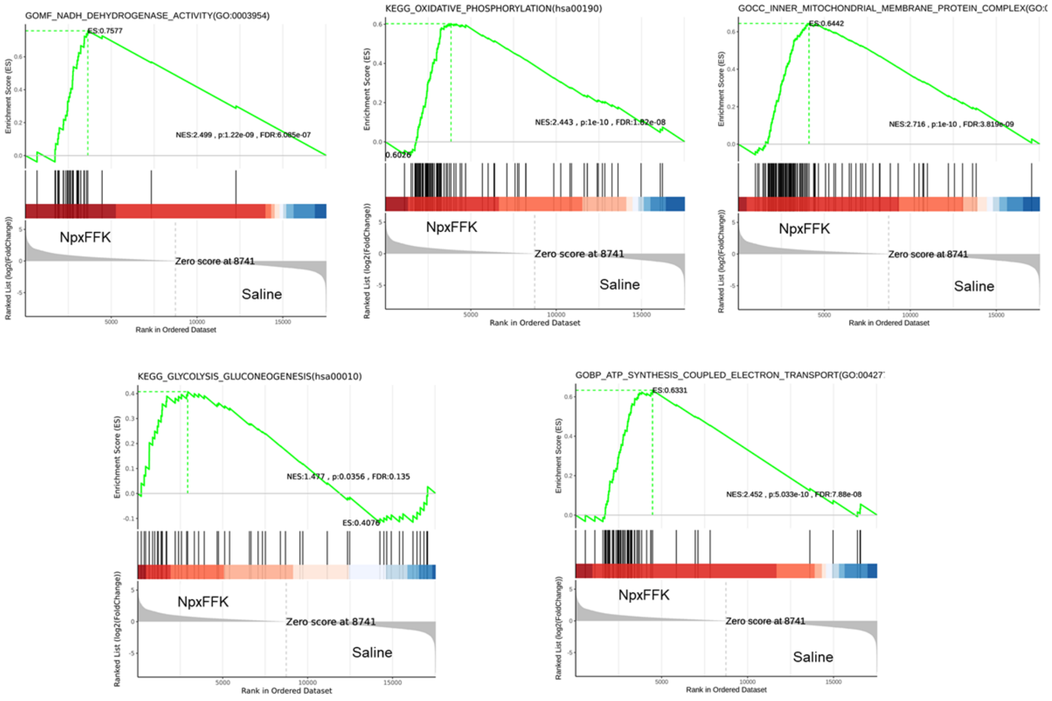

Figure S22 GESA analysis of NADH dehydrogenase activity, oxidative phosphorylation, inner mitochondrial membrane protein complex, glycolysis gluconeogenesis, and ATP synthesis coupled electron transport between the NpxFFK group and the saline group.
